# Highly Efficient Aligned Ion-Conducting Network and Interface Chemistries for Depolarized All-Solid-State Lithium Metal Batteries

**DOI:** 10.1007/s40820-023-01301-4

**Published:** 2024-01-12

**Authors:** Yongbiao Mu, Shixiang Yu, Yuzhu Chen, Youqi Chu, Buke Wu, Qing Zhang, Binbin Guo, Lingfeng Zou, Ruijie Zhang, Fenghua Yu, Meisheng Han, Meng Lin, Jinglei Yang, Jiaming Bai, Lin Zeng

**Affiliations:** 1 Shenzhen Key Laboratory of Advanced Energy Storage, Southern University of Science and Technology, Shenzhen, 518055 People’s Republic of China; 2 Department of Mechanical and Energy Engineering, Southern University of Science and Technology, Shenzhen, 518055 People’s Republic of China; 3 SUSTech Energy Institute for Carbon Neutrality, Southern University of Science and Technology, Shenzhen, 518055 People’s Republic of China; 4 Department of Mechanical and Aerospace Engineering, Hong Kong University of Science and Technology, Kowloon, 997077 Hong Kong Special Administrative Region of China People’s Republic of China; 5 HKUST Shenzhen-Hong Kong Collaborative Innovation Research Institute, Futian, Shenzhen, People’s Republic of China

**Keywords:** All-solid-state lithium metal batteries, Composite solid electrolyte, 3D printing, Areal capacity, Interfacial degradation

## Abstract

Improving the long-term cycling stability and energy density of all-solid-state lithium (Li)-metal batteries (ASSLMBs) at room temperature is a severe challenge because of the notorious solid–solid interfacial contact loss and sluggish ion transport. Solid electrolytes are generally studied as two-dimensional (2D) structures with planar interfaces, showing limited interfacial contact and further resulting in unstable Li/electrolyte and cathode/electrolyte interfaces. Herein, three-dimensional (3D) architecturally designed composite solid electrolytes are developed with independently controlled structural factors using 3D printing processing and post-curing treatment. Multiple-type electrolyte films with vertical-aligned micro-pillar (p-3DSE) and spiral (s-3DSE) structures are rationally designed and developed, which can be employed for both Li metal anode and cathode in terms of accelerating the Li^+^ transport within electrodes and reinforcing the interfacial adhesion. The printed p-3DSE delivers robust long-term cycle life of up to 2600 cycles and a high critical current density of 1.92 mA cm^−2^. The optimized electrolyte structure could lead to ASSLMBs with a superior full-cell areal capacity of 2.75 mAh cm^−2^ (LFP) and 3.92 mAh cm^−2^ (NCM811). This unique design provides enhancements for both anode and cathode electrodes, thereby alleviating interfacial degradation induced by dendrite growth and contact loss. The approach in this study opens a new design strategy for advanced composite solid polymer electrolytes in ASSLMBs operating under high rates/capacities and room temperature.

**Supplementary Information:**

The online version contains supplementary material available at 10.1007/s40820-023-01301-4.

## Highlights

- This study introduces an innovative 3D-printed electrolyte with vertically aligned ion transport network, which contains well-dispersed nanoscale Ta-doped Li_7_La_3_Zr_2_O_12_ in a poly(ethylene glycol) diacrylate matrix.
- The 3DSE architecture enables efficient ion transport across the Li/electrolyte and electrolyte/cathode interfaces, which allows for increased active material mass loading and enhanced interfacial adhesion.
- The p-3DSE Li symmetric cell displays an impressive critical current density value of 1.92 mA cm^−2^ and stable operation for 2600 h at room temperature. Full cells using p-3DSE achieve notable areal capacities.

**Supplementary Information:**

The online version contains supplementary material available at 10.1007/s40820-023-01301-4.

## Graphical abstract

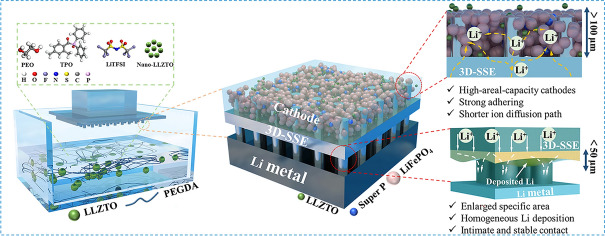

## Introduction

The advancements of electric vehicles and smart grids technologies will call for higher demands on lithium-ion batteries (LIBs) with enhanced energy density and safety features [1–3]. However, the conventional use of organic liquid electrolytes in LIBs, while showing improvements, presents inherent issues such as leakage, instability, and flammability, posing safety concerns [4]. Consequently, all-solid-state lithium metal batteries (ASSLMBs) have emerged as ideal candidates due to their high energy density, long cycle life, and especially better safety [5–7], to substitute liquid-electrolyte LIBs and address the growing demands. The energy density of ASSLMBs can be significantly enhanced by employing a lithium metal anode. This is attributed to its exceptionally low working voltage (0 V vs. Li^+^/Li) and remarkable theoretical capacity (3860 mAh g^−1^) [8–10]. Furthermore, the solid electrolytes employed in ASSLMBs are nonflammable, stable in the air (except for halides and sulfides), and less reactive with Li metal anode, indicating their suitability with highly safe Li metal batteries [11].

However, the realization of ASSLMBs is an overwhelming challenge for serious interfacial issues, both Li/electrolyte and electrolyte/cathode interfaces [12, 13]. Regarding the Li metal anode, it's important to note that the Li/electrolyte interface tends to deteriorate with repeated cycling. This degradation occurs due to the stripping and plating of lithium, which disrupts the continuous pathways for both electron and ion conduction. Consequently, this disruption leads to uneven lithium deposition and the formation of dendrites at the Li/electrolyte interface. These dendrites can further exacerbate the issue by causing a loss of interfacial contact and an increase in resistance [14]. For the cathode electrode, due to tortuous and lengthy ionic diffusion paths within electrodes, ASSLMBs typically have low active mass loading (< 1 mg cm^−2^), which is much lower than the requirements for commercial batteries, which call for 12 mg cm^−2^ for LiCoO_2_ cathode. This issue results in low energy density and poor ion transport as well as increased interfacial resistance [15, 16]. Hence, there is a strong desire to develop multifunctional electrolytes that possess several key attributes. These include excellent compatibility with lithium anodes, the capability to accommodate high mass loadings of cathode materials, the ability to establish robust interface contacts, and the provision of substantial mechanical strength.

One effective strategy to mitigate critical interface-related issues is to increase the contact area by incorporating 3D-structured components within batteries [17–19]. For Li metal, various 3D architectures (carbon-based [20], metal [21], alloy [22], etc.) featuring well-ordered micro- or nanostructures have been proven highly advantageous. These structures are instrumental in lowering local current densities, accommodating significant volumetric changes, and facilitating more uniform lithium plating and stripping. This is achieved by augmenting the electroactive surface area. Indeed, achieving high energy density in cathode materials is not as simple as merely increasing the thickness of the active materials. This approach faces challenges related to poor charge transport kinetics and compromised mechanical stability in thick electrode configurations [23, 24]. Simultaneously, electrodes featuring high mass loadings through conventional slurry-coating techniques often encounter challenges such as inadequate interfacial adhesion, sluggish chemical kinetics, and disruptions in electrical contact, all of which stem from the volumetric changes that occur during cycling [25]. Likewise, within ASSLMBs, the pursuit of both high energy density and robust safety features calls for the integration of versatile 3D architectures. These architectures are crucial for developing electrolytes that possess superior mechanical properties and exceptional ionic conductivity [26, 27]. In turn, these characteristics play a pivotal role in enhancing the overall stability of the interfaces, including the Li/electrolyte and electrolyte/cathode interfaces. Various approaches have been proposed to fabricate 3D frameworks, including template method [28], electrospinning [29], hydrogel-derived method [30] and 3D printing [15]. For example, Hu’s group [31] proposed a well-organized Li_7_La_3_Zr_2_O_12_ (LLZO) skeletons using bacteria cellulose as template, demonstrating the continuous Li^+^ transport paths and enhanced ionic conductivity. Yu’s group [30] transformed randomly dispersed Li_3*x*_La_2/3−*x*_TiO_3_ (LLTO) particles into a continuous 3D framework, and the composite polymer electrolyte delivered an ionic conductivity of 8.8 × 10^–5^ S cm^−1^ at room temperature. Bruce’s group [32] created the structural hybrid electrolytes with 3D bi-continuous ordered ceramic electrolyte and polymer matrix via the 3D printing, further exhibiting superior mechanical properties without significantly compromising ionic conductivity. Nevertheless, while previous studies have primarily focused on addressing issues such as the suppression of lithium dendrite growth and enhancement of ionic conductivity in solid electrolytes, there has been limited exploration into strategies for bolstering the interface between solid electrolytes and cathode materials, improving the reaction kinetics of cathode materials, and ultimately elevating the energy density of the entire cell. Furthermore, it's worth noting that many 3D solid electrolytes come with substantial costs and involve intricate synthesis processes, which pose challenges for mass production and practical applications. To date, there have been relatively few reports on the direct fabrication of electrolyte films that combine a 3D architecture with high ionic conductivity and excellent flexibility. This represents an area with substantial untapped potential.

Composite polymer electrolytes (CPEs), as a prominent type of electrolyte, typically showcase a combination of advantageous characteristics. These include remarkable flexibility, moderate ionic conductivity, and effective contact with electrode materials, with a particular emphasis on processability [33, 34]. Yang’s group [35] reported a 3D CPEs consist of vertically aligned Li_1.5_Al_0.5_Ge_1.5_(PO_4_)_3_ (LAGP) skeletons and polyethylene oxide (Poly(PEGDA))-based polymer matrix via simple ice template method. 2D vermiculite sheets (VAVS) were also used as raw materials to fabricate the 3D vertically aligned network by Luo’s group [36]. Inspired by the microstructure of biomass wood, Hu’s group [37] developed a vertical garnet framework by wood template, Cui’s group [38] explored AlF_3_-modified AAO with a vertically aligned structure as a 3D framework for polymer electrolytes. There have also been some other noteworthy reports on electrolytes with vertical orientations [39, 40], such as perovskite membranes with vertically aligned microchannels and inorganic-polymer nanocomposites channels [41]. Hence, the establishment of a continuous and well-structured ion-conducting network emerges as a pivotal strategy in augmenting the overall performance of ASSLMBs.

Herein, we demonstrate a novel 3D-micropatterned composite polymer electrolyte with a vertically aligned 3D ion transport network by direct 3D-printing technology, which can form a morphologically stable interface with electrode material. Specifically, the 3D-printed electrolyte slurry with adjustable viscosity and excellent rheological properties consisted of well-dispersed nanoscale Ta-doped LLZO and PEGDA matrix. The resulting 3D solid electrolytes with spiral (s-3DSE) or pillared (p-3DSE) architecture possess 3D interconnected conductive and porous frameworks, which greatly reduce the resistance and polarization during the repeated cycling process. This 3DSE architecture possesses two critical effects compared to the conventional planar CPEs. On one side, the 3DSE with the increased effective surface area can lower the local current density to retard the Li stripping and stripping at the Li/electrolyte interface, and further maintain strong contact after certain cycles. On the other side, it introduces a thick-independent effect to facilitate ion transport beyond the electrolyte/cathode interface, which improves the mass loading of active materials and reinforces the interfacial adhesion by 3D architecture. Attributing to these two effects, we demonstrate that the Li symmetric cell using a p-3DSE exhibits a high critical current density (CCD) of 1.92 mA cm^−2^ and can stably operate over 2600 h under 0.5 mA cm^−2^ at room temperature without significant interfacial degradation and early short circuit. The optimized all-solid-state Li/LFP and Li/NCM811 cells based on p-3DSE with a high mass loading of 20 mg cm^−2^ (LFP) and 22 mg cm^−2^ (NCM811) deliver a high areal capacity of 2.75 mAh cm^−2^ (LFP) and 3.92 mAh cm^−2^ (NCM811) at room temperature.

## Experimental Section

### Materials

Li_2_CO_3_ (99.9%, Sigma-Aldrich), La_2_O_3_ (99.9%, Sigma-Aldrich), ZrO_2_ (99.9%, Sigma-Aldrich), Ta_2_O_5_ (99.9%, Sigma-Aldrich), were purchased from Sigma-Aldrich. Poly(ethylene glycol) diacrylate (PEGDA, Aladdin, Mv ≈ 1000) phenylbis (2,4,6-trimethylbenzoyl)-phosphine oxide (Aladdin), succinonitrile (Aladdin), and LiTFSI (Sigma-Aldrich) were also purchased. Lithium iron phosphate (LiFePO_4_), Ni-rich layered oxides LiNi_0.8_Co_0.1_Mn_0.1_O_2_ (NCM811), conductive carbon black (Super-P), Li foil (0.45 mm, 99.9%), Al foil (20 μm, 99.99%), polyvinylidene fluoride (PVDF) were purchased from MTI Corporation. Aluminum plastic film, tab and sealing machine were purchased from Guangdong Canrd New Energy Technology Co., Ltd.

### Preparation of Nanoscale LLZTO, 3D Composite Electrolytes and 3D Cathodes

#### Preparations of the Nanoscale LLZTO Powder

The Li_6.5_La_3_Zr_1.5_Ta_0.5_O_12_ (LLZTO) garnet electrolyte was synthesized using a solid-state reaction method. Li_2_CO_3_, La_2_O_3_, Ta_2_O_5_, and ZrO_2_ were used as precursors. To compensate for Li volatilization, an excess amount of Li_2_CO_3_ by 15% was added. Raw materials with isopropanol as a solvent were mixed through ball milling at 600 rpm for 8 h with zirconium oxide balls and then dried at 80 °C for 6 h. The dried material was sintered at 900 °C for 6 h. The final sintering step was conducted at 1200 °C for 24 h. The as-synthesized LLZTO sample was ground at 600 rpm for 2 h to obtain LLZTO powders. All of the powders were stored in an Ar-filled glovebox (H_2_O < 0.01 ppm, O_2_ < 0.01 ppm).

#### Fabrication of 3D-printed LLZTO/Poly(PEGDA) Composite Polymer Electrolytes

Poly(ethylene glycol) diacrylate (PEGDA, Aladdin, Mv ≈ 1000), LiTFSI (99%, Sigma-Aldrich), self-synthesized LLZTO and photoinitiator phenylbis (2,4,6-trimethyl benzoyl)-phosphine oxide (TPO, 97%, Macklin) were mixed in weight ratios of 1.5:1:0.10:0.0075, 1.5:1:0.15:0.0075, and 1.5:1:0.20:0.0075 under a 45 °C water bath using magnetic stirring at speed of 400 rpm for overnight. The SEs were then printed with a 405 nm LED printer (Asiga Max, ASIGA) by exposing the prepared precursor 6 s at a power of 9 W cm^−2^ with a layer thickness of 50 μm in a humidity below 30%, under the designed geometry (three types, including planar, spiral and pillar structures). Anhydrous ethanol (99.7%, Energy Chemical) was then used to wash away the uncured precursor. Fully photopolymerized CPEs were dried in a vacuum oven at 25 °C for 2 h, followed by further drying in an Ar-filled glovebox with < 0.01 ppm H_2_O level for at least 48 h. After the printing process, three-type composite polymer electrolytes were successfully fabricated in a direct 3D printing technology.

#### Preparation of LFP/3DSE and NCM811/3DSE Cathodes

The cathode electrodes were prepared by mixing LFP or NCM811 powder (70 wt%), super-P (10 wt%), and CPE (20 wt%, as the binder) in acetonitrile and vigorously stirred overnight. The slurry was then sprayed onto three-type CPEs, followed by drying in vacuum ovens at 30 °C overnight and further drying in an Ar-filled glovebox (H_2_O < 0.01 ppm, O_2_ < 0.01 ppm) for at least 24 h. In these experiments, LFP/3DSE and NCM811/3DSE composite cathodes with different mass loadings were obtained by controlling the spray time from 5 to 30 min, thus achieving integrated cathode/electrolyte varying the thickness between 20 and 160 μm.

## Results and Discussion

### 3D Printing Fabrication 3DSE

**Fig. 1:**
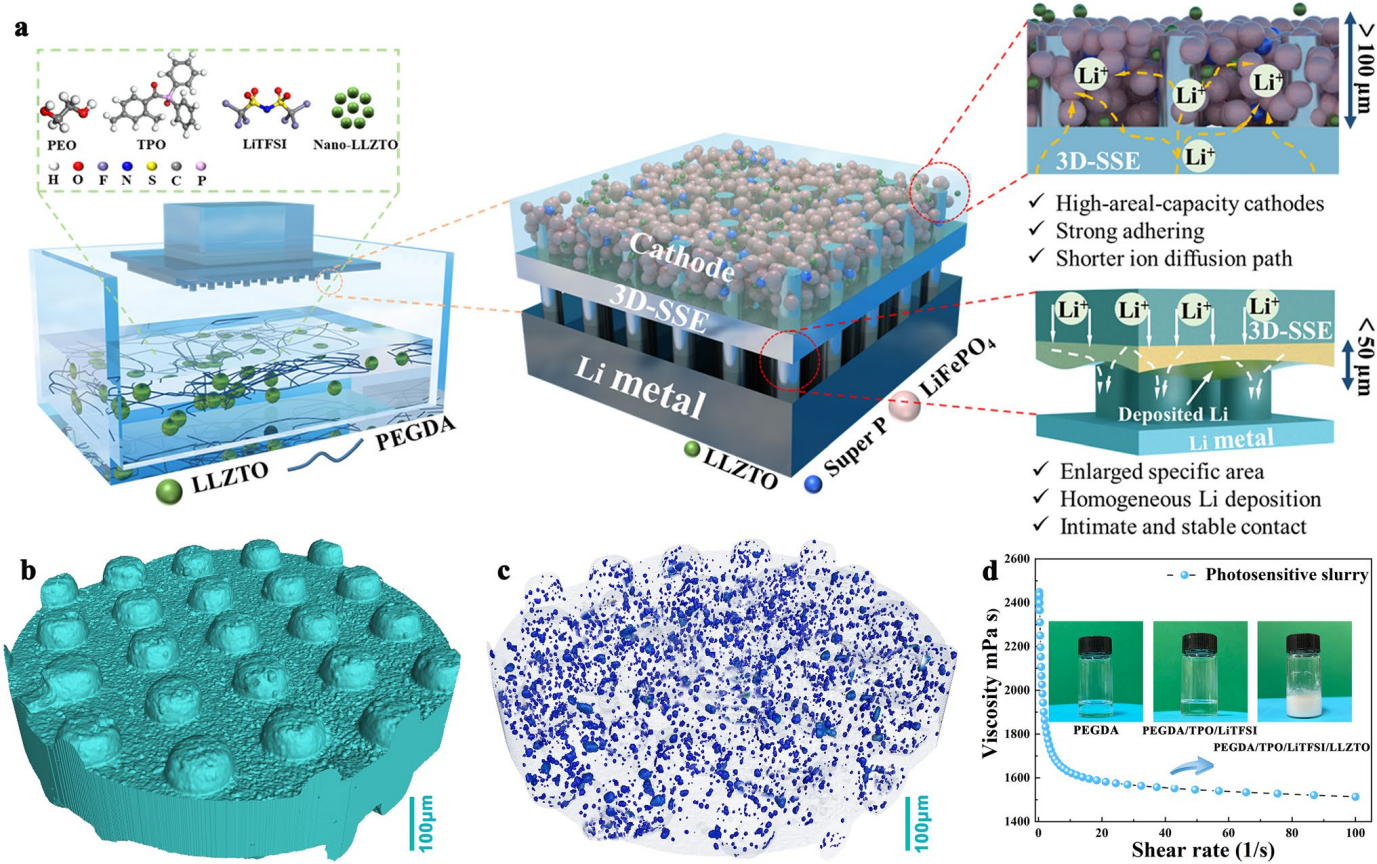
**a** Schematic illustration of the fabrication of 3D printing 3DSE and structure-property relationships. **b** Micro-CT image of the symmetric p-3DSE and **c** distribution of nano-LLZTO ceramic electrolyte in 3D architecture. **d** The viscosity of the as-obtained printed slurry (The insets are optical pictures of the slurry at different stages)

The digital light processing (DLP) 3D printing fabrication of 3DSE, including the nanoscale Li_6.5_La_3_Zr_1.5_Ta_0.5_O_12_ (nano-LLZTO), lithium bis(trifluoromethane sulfonyl)imide (LiTFSI), polyethyleneglycol diacrylate (PEGDA 1000) monomer, and photoinitiator (TPO) is schematically illustrated in Fig. 1a. The spiral and pillar-structured 3DSE were designed and fabricated in this study. PEGDA monomers were crosslinked via precise light exposure and then produced Poly(PEGDA) polymer electrolyte with high ionic conductivity. Specifically, PEGDA monomer with 0.5 wt% TPO, the inorganic electrolyte (nano-LLZTO), and lithium salt (LiTFSI) with different weight ratio was mixed (Fig. S1). A planar substrate was printed first, followed by a spiral structure of a vertically oriented cylindrical structure. More details can be found in Materials and Methods part in supporting information. After a two-step curing process, 3D composite electrolytes with symmetrical structures on both sides were obtained, whose structural factors were independently controlled. During printing, the 405 nm wavelength LED was projected to the resin surface according to the designed model, followed by a layer-by-layer stacking process. The obtained 3DSE with rational design affords critical advantages for cathode active materials and Li metal anode. For the cathode materials, vertically aligned 3DSE with low tortuosity provides short and direct ion transport pathways and mechanical support due to their highly ordered structure and channels; high mass loading of cathodes can be achieved due to the 3D space, which contributes to high energy and power density; and strong adhering of active materials and highly efficient ion transport network enable fast kinetics and stable contact interfaces. For the Li anode, enlarged specific area can promote uniformly Li plating/stripping; polymer network with uniformly distributed inorganic electrolyte powder enables a high ionic conductivity; and the remarkable mechanical properties play a vital role in suppressing Li dendrite growth and ensure the structural integrity after cell assemble. As a result, the micro-CT image of the p-3DSE that assembled by curing two pieces of the printed parts, shows the structural characteristics of the symmetrical vertically aligned pillars (Fig. 1b) and distribution of nano-LLZTO ceramic electrolyte in 3D architecture (Fig. 1c), which contributes to the enhanced ion conductivity. Figure 1d shows the viscosity of the composite printing slurry, which exhibit a typical shear thinning phenomenon. The viscosity of the slurry is about 2000 cps at shear rate of 1/s, which provides a suitable release force during printing process and guarantee the completeness of the microstructures. The insets are optical pictures of the slurry at different stages.

### Structure and Properties Characterizations

**Fig. 2:**
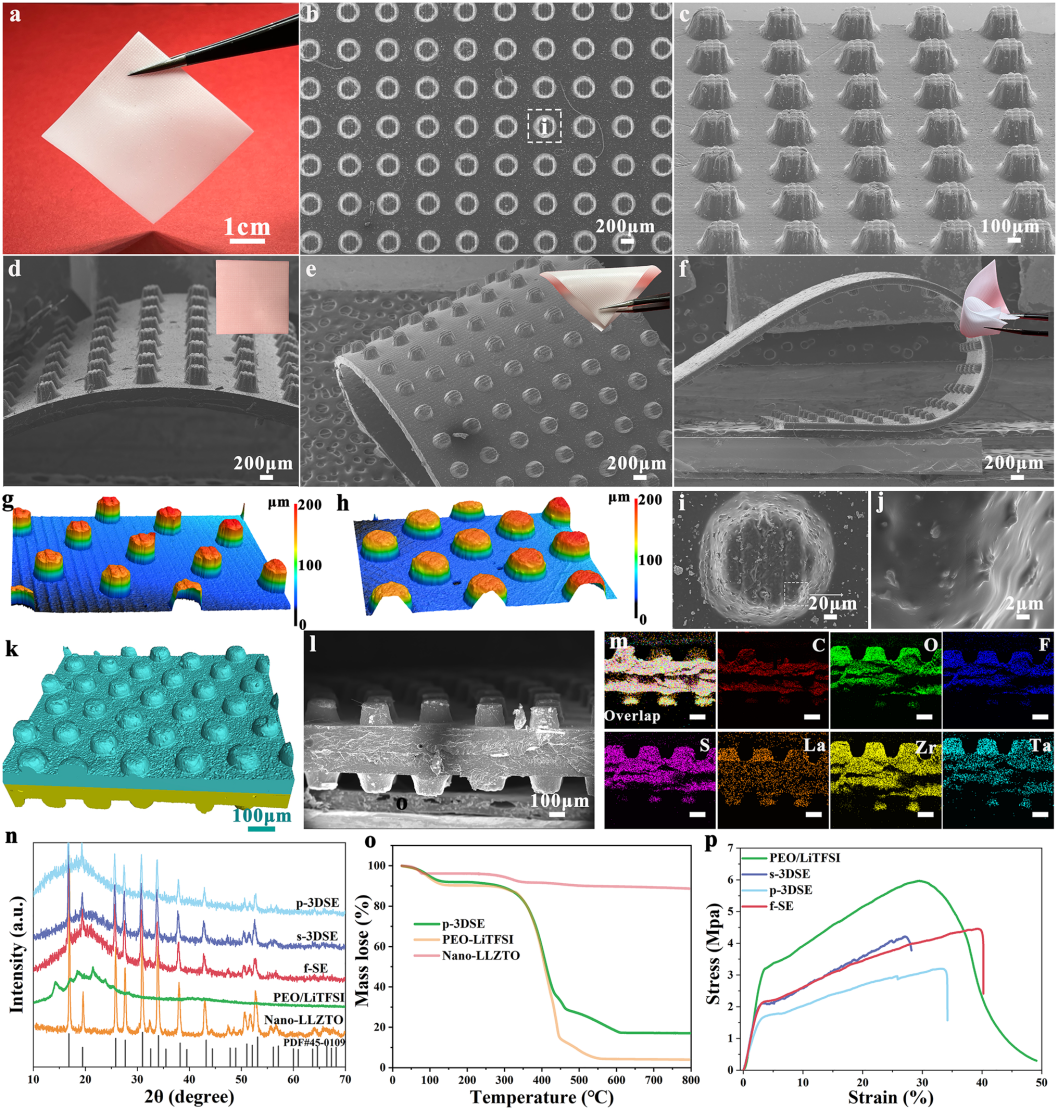
**a** Optical images and SEM images of **b, i, j **top and **c** front view of p-3DSE. SEM images of p-3DSE electrolyte at different states of **d** free, **e** bending, and **f** twisting (The inset images are optical images of large-scale p-3DSE film under different states). **g, h** Typical LCSM images of p-3DSE with different sizes. **k** Micro-CT image and l SEM image of cross-sectional and m corresponding EDS mapping data of p-3DSE. **n** XRD patterns, **o** TGA curves and **p** stress-strain curves of various electrolyte examples

The various 3DSE were successfully prepared via the 3D-printing method, and its detailed characterization results can be seen in Fig. 2. Based on the above-mentioned printable slurry, we first printed two kinds of electrolyte with 3D architecture, including 3D spiral structure (s-3DSE) and 3D pillared structure (p-3DSE). Figure 2a shows a flexible electrolyte film with large-size, which can be made of PEGDA matrix and nanosize LLZTO electrolyte. The planar structure served as the control group (noted as f-SE, Fig. S2). A series of optical images demonstrated that 3D-printed electrolytes possess excellent flexibility and good mechanical strength for f-SE, s-3DSE and p-3DSE, including bending, twisting and rolling repeatedly (Fig. S3). The inorganic electrolyte LLZTO used in these electrolytes was prepared by a high-energy ball mill. The particle size distribution was 200–500 nm from SEM images (Fig. S4) and TEM images (Fig. S5), which is beneficial to evenly disperse in EO and improve ionic conductivity. Scanning electron microscopy (SEM) images further exhibited 3D architectural structural details of the printed electrolyte. The p-3DSE duplicated the designed geometry with a pillared structure of 100 μm width, 150 μm height, and 200 μm spacing on a planar substrate of 75 μm thickness (Fig. 2b, c). Figure 2d–f exhibits good flexibility and functionality at different states of free, bending, and twisting for p-3DSE electrolyte. The insets are optical images of large-sized p-3DSE electrolyte films under different bending states. The enlarged SEM images revealed the morphologies of a single pillar and its surface, which shows the complete vertically aligned framework and smooth surface features (Fig. 2i, j). The same characteristics occurred in both planar electrolytes and s-3DSE electrolytes from SEM images (Figs. S2c, d and S6). The thickness of the plane electrolyte is 75 μm, which is consistent with the design thickness (Figs. S2a and S6a, e). The s-3DSE with the spiral microstructure of 100 μm width and 150 μm height was also successfully printed, which formed a continuous support structure (Fig. S6c, d). The flat morphology of the s-3DSE surface and planar structure indicates that the LLZTO remained immobilized in the structure of the PEGDA matrix. In order to further improve the effective contact area between the electrolyte surface and electrodes, we have further optimized the vertically oriented pillared structure. From 3D and 2D laser scanning confocal microscopy (LSCM) images, vertically oriented 3D electrolytes with different sizes can be visually compared, especially with respect to the diameter and density of the pillars (Figs. 2g, h and S7). In detail, a series of p-3DSE with different sizes were obtained by varying the height (50, 100, 150, 200 μm) and diameter (200, 300 μm) of the pillars, with the spacing between the pillars remaining constant at 200 μm throughout the printing process. The thickness of the planar substrate was maintained at 75 μm (Fig. S8).

The cross-sectional morphology of the symmetrical p-3DSE was evaluated by the micro-CT and SEM. As shown in Fig. 2k, l, the p-3DSE films could keep flat after the post-curing process, which is vital for ASSLMBs assembly. The EDS mappings in Fig. 2m reveal that the p-3DSE was successfully integrated with the PEGDA, LiTFSI and LLZTO. X-ray diffraction (XRD) patterns of the various electrolytes are shown in Fig. 2n. The diffraction patterns match well with the standard pattern of LLZTO ceramics electrolyte, which indicates well-maintained CPEs after incorporating with the PEGDA matrix. Thermogravimetric analysis (TGA) curves are shown in Fig. 2o to evaluate the thermal stability of the CPEs. It is clarified that the p-3DSE exhibits enhanced thermal stability due to the introduction of nano-LLZTO. The mechanical properties of the obtained 3D electrolytes were characterized by the tensile test at room temperature. The p-3DSE exhibited high mechanical strengths of 3.25 MPa, which is robust enough for using in coin cells and pouch cells (Fig. 2p). The introduction of nano-LLZTO and vertically aligned pillared structure does not greatly sacrifice the mechanical properties of electrolyte, which was ascribed to the good adhesion between the PEGDA matrix and nanoscale LLZTO ceramic.

**Fig. 3:**
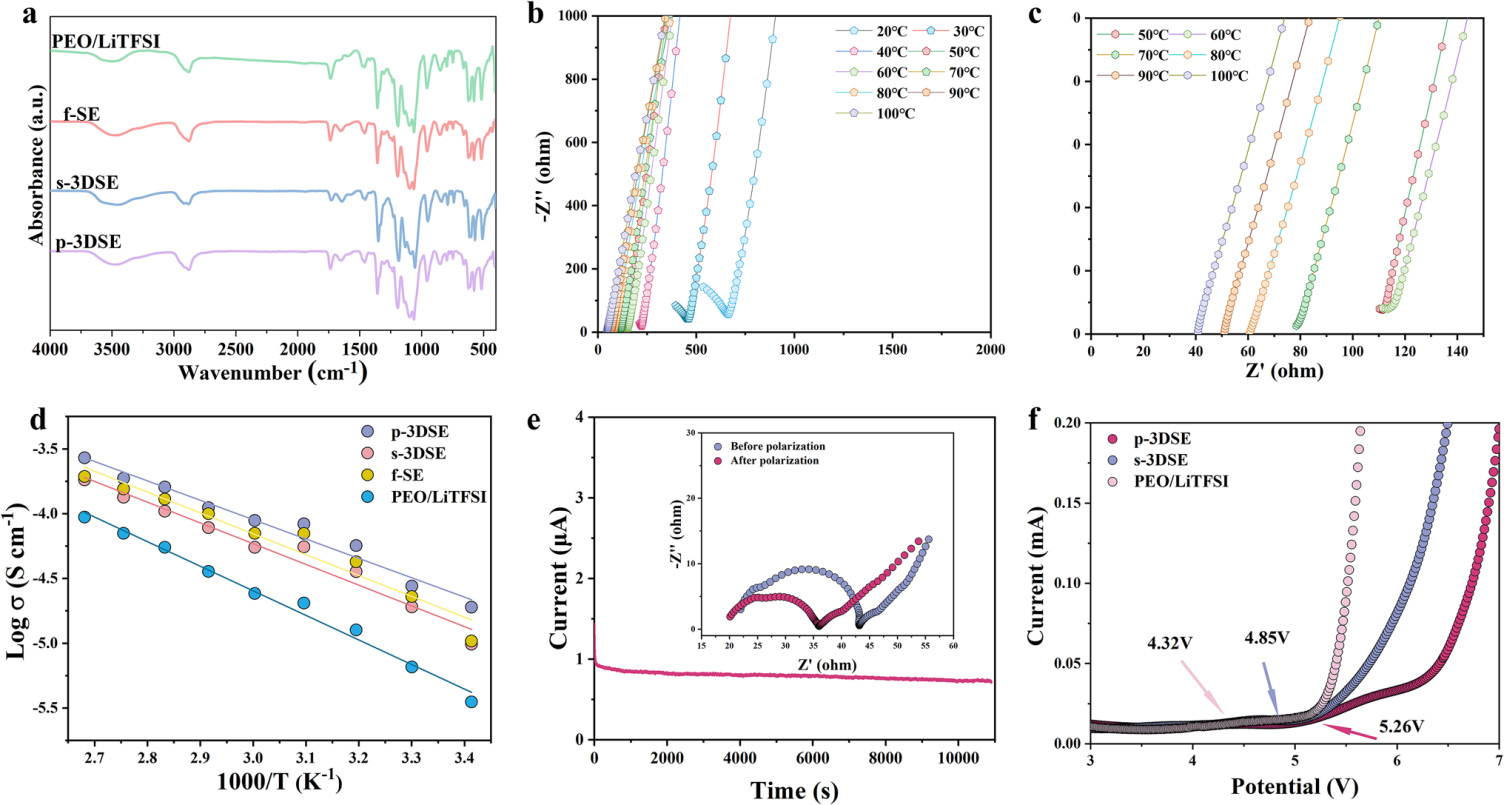
**a** FTIR spectra for Poly(PEGDA)/LiTFSI electrolyte, f-SE, S-3DSE and p-3DSE electrolytes. **b**, **c** Impedance spectra for p-3DSE electrolytes from 20 to 100 °C. **d** Arrhenius plots of p-3DSE electrolyte. **e** Chronoamperometry curves of p-3DSE electrolytes (the inset image is the EIS curves of p-3DSE before and after polarization) and **f** LSV curves of Poly(PEGDA)/LiTFSI, s-3DSE, and p-3DSE electrolytes

Fourier transform infrared (FTIR) were performed to investigate the interaction between the nano-LLZTO ceramic and Poly(PEGDA) matrix (Fig. 3a). Poly(PEGDA)-based electrolytes clearly display the typical bands of C–O–C stretching (1132, 1086, 1053, and 952 cm^−1^), attributing to the ether oxygen in PEGDA, as well as S=O stretching (654 cm^−1^) and LiTFSI aggregation (1634 cm^−1^) attributing to the Li salts. The peak for LiTFSI aggregation in the s-3DSE electrolyte shifts from 1634 to 1654 cm^−1^ when compared to the Poly(PEGDA) electrolyte, and its intensity decreases. This is also consistent for the p-3DSE electrolyte, which suggests that the addition of LLZTO garnet ceramic electrolyte can promote the LiTFSI dissociation and thus free more Li^+^. The Li ionic conductivity of as-obtained electrolyte films was determined by electrochemical impedance spectroscopy (EIS) using symmetric stainless steel/electrolyte/stainless steel (SS/electrolyte/SS) cells. The Nyquist plots of the SS/p-3DSE/SS cell in a temperature range of 20–100 °C are shown in Fig. 3b. The enlarged Nyquist plot of SS/p-3DSE/SS cell at the high-frequency region is shown in Fig. 3c. It is obvious that the resistance of the p-3DSE is much lower than that of other 3D-printed electrolytes (Figs. S9 and S10). Temperature-dependent ionic conductivity (*σ*) curves (Fig. 3d) reveal that the ionic conductivity can be improved with elevated temperature. Compared with the Poly(PEGDA)/LiTFSI solid electrolyte, the conductivity of the p-3DSE is as high as 3.15 × 10^–4^ S cm^−1^ at 30 °C, which meets the room temperature requirements of ASSLMBs. The *σ* of p-3DSE reaches 1.05 × 10^–3^ S cm^−1^ at 60 °C, which is two orders of magnitude higher than Poly(PEGDA)/LiTFSI electrolyte (2.41 × 10^–5^ S cm^−1^) at the same temperature. The activation energy (*E*_a_) of the p-3DSE (0.25 eV) is lower than that of the s-3DSE (0.36 eV), f-SE (0.49 eV) and Poly(PEGDA)/LiTFSI electrolyte (0.70 eV), indicating a low activation barrier for the dissociation of ion pairs and local ion hopping. The Li-ion transference number (*t*_Li_^+^) of p-3DSE, s-3DSE, f-SE, and Poly(PEGDA)/LiTFSI electrolyte is 0.68, 0.61, 0.42, and 0.34, respectively (Figs. 3e and S11). Obviously, the *t*_Li_^+^ of all printed electrolytes with LLZTO ceramic is higher than that of the Poly(PEGDA)/LiTFSI electrolyte. In particular, p-3DSE possesses an optimized Li-ion transference number of 0.71, which is ascribed to the 3D architecture with vertically aligned lithium-ion channels and the interaction between the LLZTO particles and Poly(PEGDA) matrix (Table S1). Linear sweep voltammetry (LSV) curves clearly show the current value of Poly(PEGDA)/LiTFSI electrolyte suddenly rising at 4.32 V, indicating that the electrolyte has begun to decompose, while the p-3DSE and s-3DSE maintains a stable value until approximately 5.26 and 4.85 V, indicating that the printed 3DSEs are more capable of supporting high-voltage cathodes. These results demonstrate that the 3D-printed p-3DSE with low tortuosity and low activation energy can contribute to lowering the overpotential by promoting the Li^+^ transport within the electrodes.

### Electrochemical Performance

**Fig. 4:**
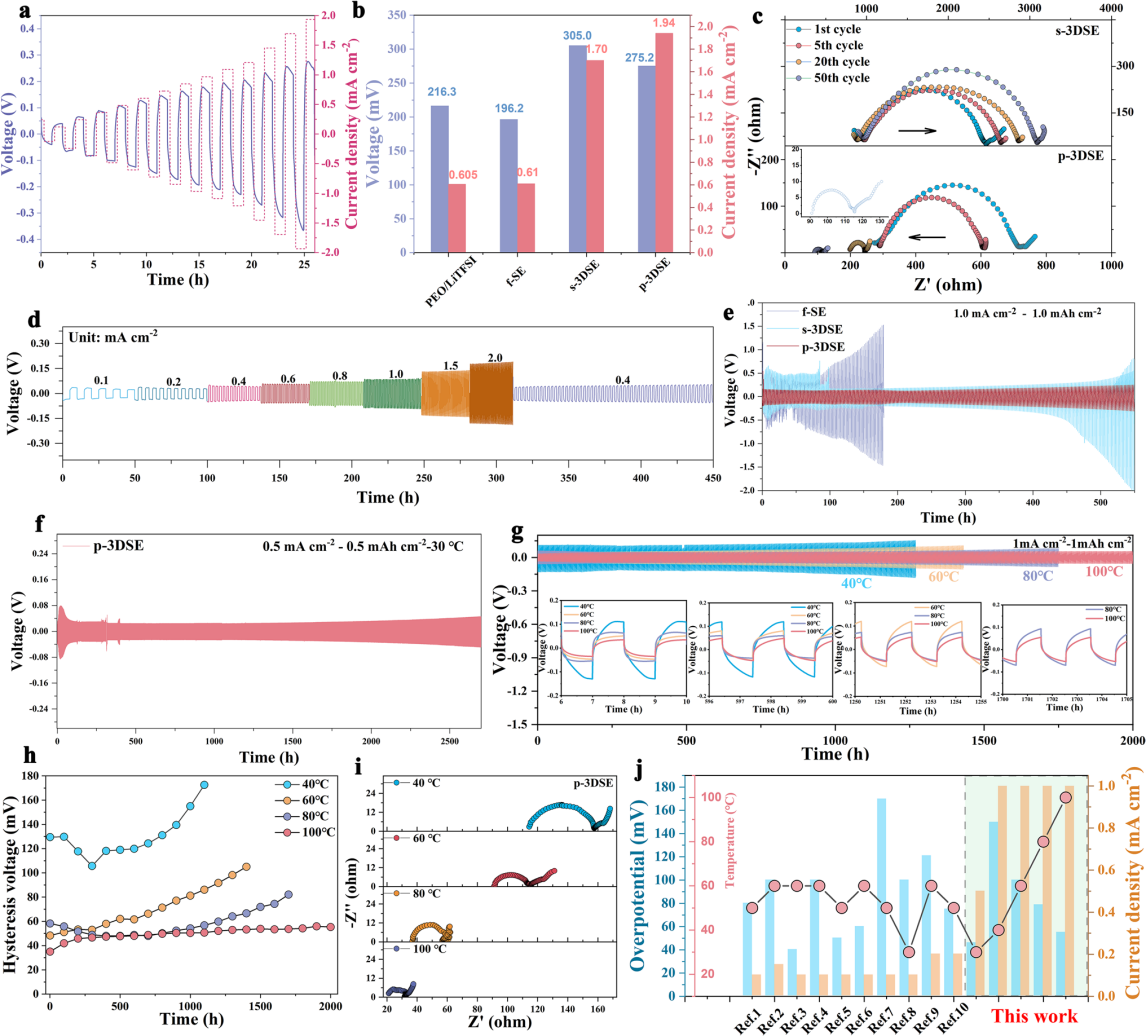
**a** CCD testing on the Li/p-3DSE/Li cells with current steps from 0.05 to 2.0 mA cm^−2^. **b** Comparison of the CCD of various electrolytes. **c** EIS spectra of the Li/f-SE/Li and Li/p-3DSE/Li cells cycled after different cycles. The symmetrical cells performance** d** rate capability, cycling **e** at 30 °C under 1 mA cm^−2^/mAh cm^−2^ and **f** 0.5 mA cm^−2^/mAh cm^−2^. **g** Long-term cycling of Li/p-3DSE/Li cells under varying temperatures from 40 to 100 °C and **h** the corresponding tendency of polarization voltage. Insets in **g** show an enlarged view of the voltage profiles in 6–10 h, 596–600 h, 1250–1255 h, and 1700–1705 h. **i** EIS spectra of the Li/p-3DSE/Li cells at different temperatures. **j** Comparison of the cycling current density and lifespan of Li symmetric cells with previously reported 3D electrolytes

The critical current density (CCD) testing with Li/p-3DSE/Li, Li/s-3DSE/Li, Li/f-SE/Li, and Li/Poly(PEGDA)/LiTFSI/Li cells was performed first. During the testing, the current density increased stepwise from 0.2 to 2.0 mA cm^−2^. As shown in Fig. 4a, the CCD for p-3DSE, s-3DSE, f-SE and Poly(PEGDA)/LiTFSI electrolyte is 1.92, 1.32, 0.95, and 0.61 mA cm^−2^, respectively (Fig. S12). For p-3DSE, even at a high current density of 1.92 mA cm^−2^, a polarization voltage of 275 mV is obtained (Fig. 4b), which is indicative of the improved CCD performed as compared with other reported electrolytes [42–46] This reveals that 3DSE with highly ordered microstructure can lower the local current density and suppress Li dendrite growth at the Li/3DSE interface. The EIS spectra for the Li symmetrical cells with p-3DSE, s-3DSE, f-SE and Poly(PEGDA)/LiTFSI electrolyte after different cycles are shown in Figs. 4c, S13 and Table S2. The reduced semicircle in the low-frequency region shows that the charge transfer resistance (*R*_ct_) decreases ascribed to the gradually optimized Li/electrolyte interfaces after repeated cycling. In addition, the starting point of the high-frequency region also decreases gradually, indicating the reduction of the bulk resistance of the solid electrolyte (*R*_s_). In contrast, the cells with Poly(PEGDA)/LiTFSI electrolyte exhibits an increasing interface resistance from 1st cycle to the 50th cycle, corresponding to 1762.0 and 2160.0 Ω. Interestingly, the Li/p-3DSE/Li cells reveal a lower initial interface resistance of 287.8 Ω compared with Li/Poly(PEGDA)/LiTFSI/Li cells, while the interface impedance is only 199.2 Ω after 20 cycles of activation, especially after 50 cycles, the interface impedance maintained to be 90.97 Ω. The excellent interfacial compatibility of the Li/p-3DSE/Li cells can be attributed to the 3DSE with enlarged surface areas and highly efficient ion transport channels.

Meanwhile, the Li/p-3DSE/Li cells deliver excellent rate performance in the symmetric cell at room temperature (Fig. 4d). In detail, the cells show low voltage polarizations of 55, 65, 75, 86, 100, 115, 124, and 150 mV at current densities of 0.1, 0.2, 0.4, 0.6, 0.8, 1.0, 1.5, and 1 mA cm^−2^, respectively. However, the Li/ Poly(PEGDA)/LiTFSI/Li cells and Li/f-SE/Li cells display more serious voltage polarizations, especially at high current densities (Fig. S14). The Li/p-3DSE/Li cells remain stable within 600 h of cycling under room temperature at 1 mA cm^−2^ and a capacity of 1 mAh cm^−2^ (Figs. 4f and S15). No short circuit occurs after 600 h and the polarization voltage remains stable at approximately 80 mV. This indicates good interface stability between the p-3DSE and Li metal. However, the Li/s-3DSE/Li cells assembled with the f-SE exhibit abrupt changes in voltage only within 50 h, and the cells show significant initial voltage changes and subsequent stabilization as well as the increasing voltage over 400 h of Li plating/stripping, demonstrating the continuous growth of Li dendrites during uneven Li plating/stripping process. Figure 4f shows the long-term cycling performance of Li/p-3DSE/Li cells at room temperature. At a current density of 0.5 mA cm^−2^, the cells are stable, and continuously cycled beyond 2600 h without short-circuiting, which indisputably demonstrates an exceptional cycling stability. Furthermore, the Li/p-3DSE/Li cells demonstrate outstanding cycle capability under different temperatures from 40 to 100 °C, and showed excellent long-term cycling stability beyond 1250, 1400, 1750, and 2000 h, respectively (Fig. 4g). The insets detailed exhibit the plating/stripping curves of the cells with different electrolytes at different stages. The voltage hysteresis curves clearly indicated that the Li/p-3DSE/Li cells exhibited the most stable polarization voltage and interface stability (Fig. 4h). The EIS profiles under different temperatures after certain cycles at 1 mA cm^−2^ are shown in Fig. 4i, indicating fast ion transport and a stale solid–solid interface. These results present significant improvements in terms of CCD and long-lifespan under room temperature among the previously reported 3D electrolytes in terms of cycling lifespan and current density (Fig. 4j, Tables S4).

**Fig. 5:**
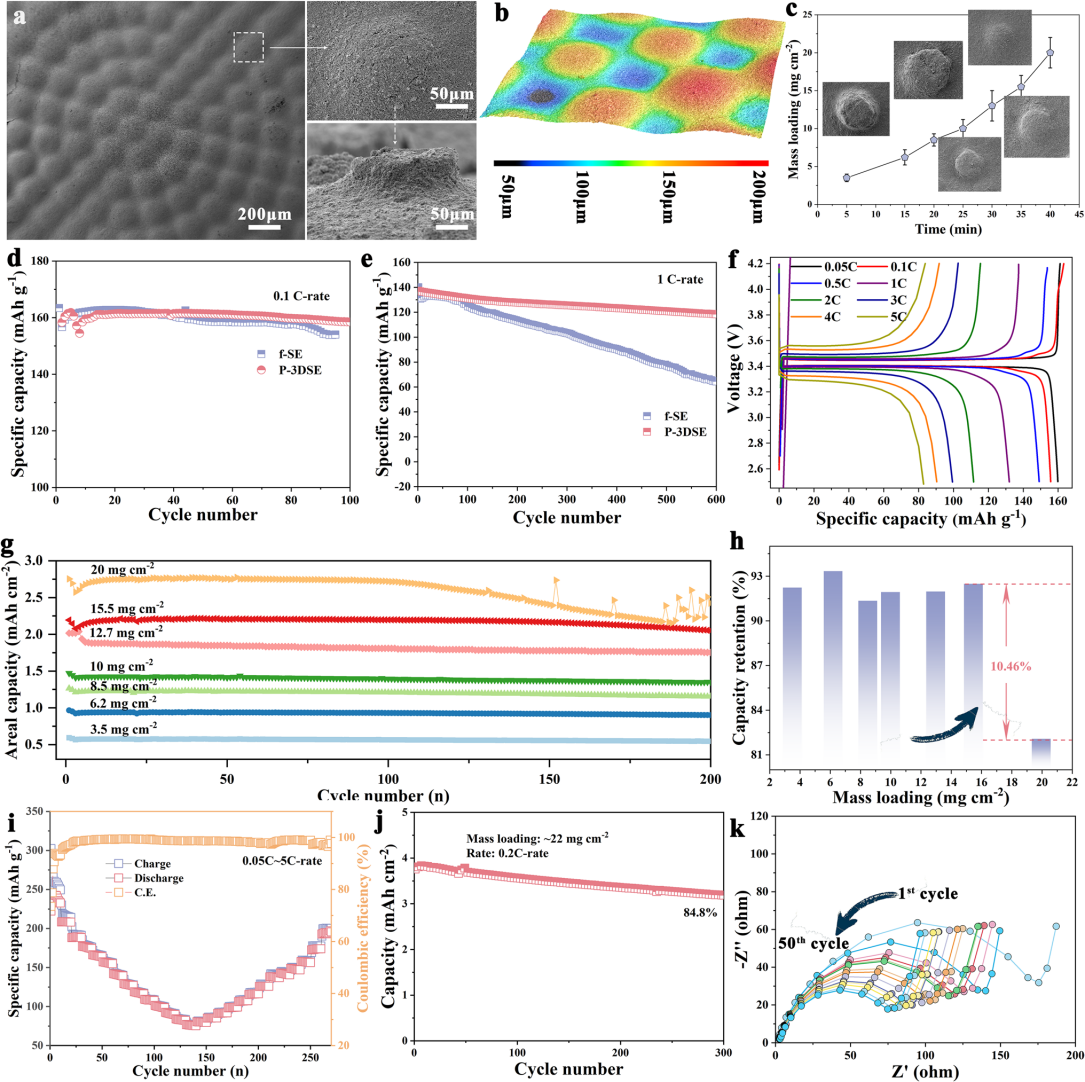
**a** SEM images of p-3DSE/LFP cathode from the top and cross-sectional views. **b** LCSM images of p-3DSE/LFP cathode. **c** The curve of mass loading of LFP cathode and spraying time. Cycling performance of cells using LFP cathode at **d** 0.1C, **e** 1.0C, and **f** typical charge/discharge profiles at various rates. **g** Cycling life of LFP cathode with different mass loadings and **h** the corresponding capacity retention. **i** Rate performance, **j** cycling lifespan under high mass loading and **k** EIS spectra of Li/p-3DSE/NCM811 cells

In ASSLMBs, electrodes under high mass loadings, to enhance areal capacities, are necessary. However, sluggish ions transport and poor electrode flexibility could occur when blindly increasing the thickness of the electrode. In this regard, we skillfully designed 3D electrolyte architectures, especially for cathode materials. SEM images indicate the optimized p-3DSE/LFP electrodes by a simple spraying process (Fig. S16). It is obvious that the LFP material is uniformly and firmly attached to the 3D electrolyte frameworks, as shown in Fig. 5a. The enlarged SEM images from the top and cross-sectional show a smooth surface and strong contact. Through the LCSM image, it can be further proved that the LFP active materials are uniformly distributed in the 3D electrolyte skeletons and remain over 100 μm thickness (Fig. 5b). By controlling the spraying time, the cathode electrodes with different mass loadings can be prepared (Fig. S17). In particular, a composite p-3DSE/LFP electrode with a loading beyond 15 mg cm^−2^ can be obtained (Fig. 5c). Strong adhesion between cathode material and p-3DSE can be found through the cross-sectional SEM images (Fig. S18a, b), compared with the electrodes fabricated by the traditional method (Fig. S18c, d). In order to investigate the relationship between the thickness of active materials and ion transport, two commercial materials, LFP and NCM811, were selected as the cathode materials, and a series of thickness electrodes were fabricated. The galvanostatic charge–discharge of Li/p-3DSE/LFP and Li/f-SE/LFP full cells at 0.1C-rate from 2.5 to 4.2 V was performed. As shown in Fig. 5d, after 100 cycles, there is a capacity retention of 97% for the Li/p-3DSE/LFP cells. However, the capacity retention of the Li/f-SE/LFP cell drops to 83.1%. While the cycling stability of Li/p-3DSE/LFP is improved, a capacity retention of 89% after 600 cycles at 1C-rate is obtained (Fig. 5e). Figure 5f illustrates the rate performance under different rates (Fig. S19), and the Li/p-3DSE/LFP cells show improved cyclic stability and rate performance than the Li/ Poly(PEGDA)/LiTFSI/LFP (Fig. S20). The Li/p-3DSE/LFP cells assembled with different mass loadings exhibit superior cycling capacities, indicating that high Li-ion transport expressways are formed in the cells. Figure 5g indicates the cycling performance of Li/p-3DSE/LFP cells at 0.5C-rate under different loadings. With the mass loadings of 3.5, 6.2, 8.5 10, 12.7, 15.5, and 20 mg cm^−2^, the reversible capacities of these cells are 0.576, 0.94, 1.227, 1.426, 1.872, 2.203, and 2.754 mAh cm^−2^, respectively. This result indicates that p-3DSE with enlarged surface area and high ion-conducting frameworks can still maintain stability, provide Li^+^ transport pathways and improve the contact between the electrolyte and electrode. After 300 cycles, the Li/p-3DSE/LFP cells with the mass loading of 15.5 mg cm^−2^ possess a higher capacity retention of 92.48% compared with the Li/p-3DSE/LFP cells with the loading of 20 mg cm^−2^, indicating that interface failure may occur when the electrode thickness exceeds 150 μm. Moreover, the Li/p-3DSE/NCM811 cells exhibit excellent rate performance from 0.05C-rate to 5C-rate, as shown in Figs. 5i and S21. The Li/p-3DSE/NCM811 cells provide capacities of 208.3, 189.2, 156.8, 108.3, and 100.0 mAh g^−1^ at 0.1, 0.2, 0.5, 1, and 2C-rate, respectively. Even at 5C-rate, the cell still delivers a reversible capacity of 76.23 mAh g^−1^. The long-term cycling performance of the Li/p-3DSE/NCM811 cells under the loading of 22 mg cm^−2^ at 0.2C-rate is shown in Fig. 5j, and the high reversible capacity of 3.19 mAh cm^−2^ can be obtained even after 300 cycles with a high capacity retention of 84.8%, which is ascribed to lower interfacial resistance and shorter ion transport pathway within electrodes. The EIS spectra of Li/p-3DSE/NCM cells after different cycles were tested. As shown in Fig. 5k, it is noted that the semicircle gradually decreases as the cycle number increases, indicating an activation process between the electrolyte/electrode interfaces in initial cycles and enhanced Li^+^ ion transfer. The stable electrolyte/electrode interfaces can be established over time (Table S5). To verify the practicality of 3D printing electrolytes, the LFP/Li pouch cells were assembled to test the practicality of the p-3DSE electrolyte in ASSLMBs. The LFP/Li cells can light on the LED under bending, folding, and after recovery (Fig. S22), indicating good flexibility and functionality at different states of the 3D-printed p-3DSE electrolyte (Fig. 2c–f).

### Interfacial Evolution of Morphologies and Chemistries

**Fig. 6:**
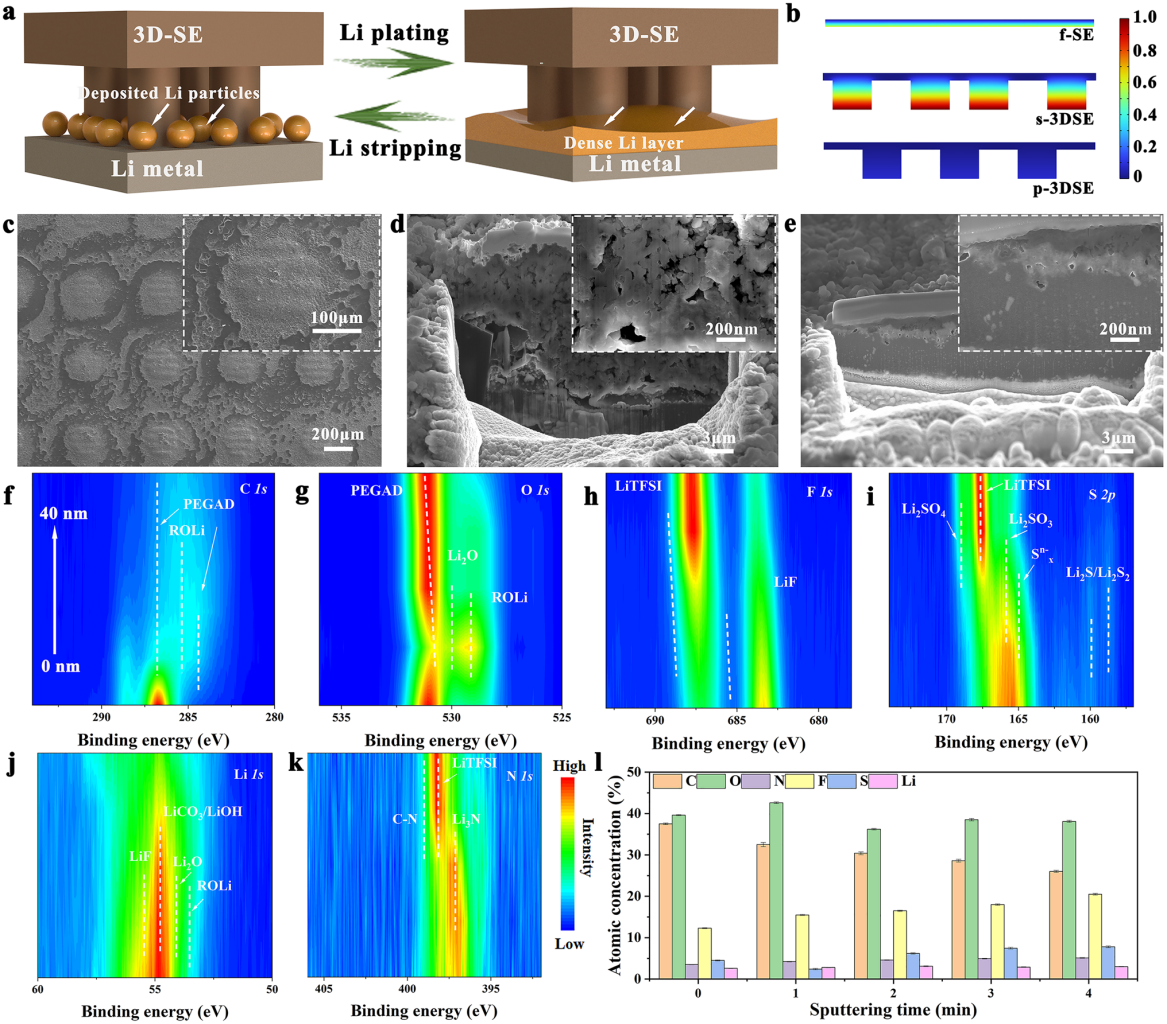
**a** Schematic illustration of the plating process of Li metal with the p-3DSE. **b** Li^+^ concentration distribution ascends along the Li anodes interface and three-type electrolytes based on simulation. SEM images of **c** p-3DSE after cycles from top-view, Li metal cycled beyond 1000 times under **d** f-SE electrolyte and **e** p-3DSE electrolyte after FIB cutting from cross-sectional view (Insets are enlarged SEM images). Sputter-down XPS spectra of **f** C 1*s*, **g** O 1*s*, **h** F 1*s*, **i** S 1*s*, **j** Li 1*s*, and **k** N 1*s* spectra of p-3DSE interfaces. **l** Bar charts showing the atomic concentrations of different sputtering times in the electrolyte interface

The morphological evolution at Li/SE, Li/f-SE, and Li/p-3DSE interfaces during galvanostatic cycling under 0.1 mA cm^−2^ (0.5 mAh cm^−2^) was tracked by post-mortem analysis. A schematic illustration was established to understand the electrolyte/Li interface interaction (Fig. 6a). In Li/electrolyte/Li cells, significant damage of the interface occurred in less than 50 h for the bare Poly(PEGDA)/LiTFSI electrolyte, and pores and voids were extensively detected not only at the surface of Li metal (Fig. S23) but also in the solid electrolyte film (Fig. S24). In contrast, the Li/p-3DSE/Li cells steadily maintained the stripping and deposition processes due to increased effective active area with Li. The nucleation and penetration of Li dendrites from the interface can be efficiently suppressed, as confirmed by the clean SEM images of the cycled p-3DSE at different stages. At the initial stage of cycles, the pillar arrays of p-3DSE were still clearly visible due to the small-capacity Li deposition (Fig. S25a). The enlarged SEM images show that the Li metal is uniformly and tightly deposited on the surface of the electrolyte pillar. A dense and smooth Li/electrolyte composite was formed within 20 h when the capacity of Li increased with cycling, indicating a stable and robust interface (Figs. S25b and 6c). The surface of Li metal with p-3DSE electrolyte also showed uniform and dense Li, without Li dendrite growth (Figs. 6e and S26), compared with the f-SE (Fig. 6d). From the optical images, the Li metal after cycles was flat and shiny (Fig. S27a). In contrast, the surface of lithium metal with bare Poly(PEGDA)/LiTFSI electrolyte showed black spots, and massive dendrites and pits (Fig. S27b). The p-3DSE film also delivered dense and smooth morphology after repeated cycles (Fig. S28). The results show serious interface degradation in the Li/SE and Li/f-SE cells. However, the Li flux toward the interface can become sufficient to replenish the Li loss at p-3DSE areas, which prevents interfacial degradation. Multiphysics simulation was conducted to monitor ions distribution at the Li/electrolyte interfaces (Fig. S29). As shown in Fig. 6b, the Li symmetric cell with p-3DSE electrolyte possesses low Li^+^ concentration gradient, which clarifies fast ion conduction and reaction kinetics occurred at the Li/p-3DSE interface tended toward a uniform Li deposition, instead of forming dendritic Li at initial lithium plating stages. Nevertheless, severe Li^+^ concentration gradient and fluctuations can be observed at the Li/f-SE and Li/s-3DSE interfaces, meaning that unavailing Li^+^ depletion or Li dendrites would occur at interfaces due to the locally fluctuated Li^+^ flux.

In order to probe the reasons for the good compatibility of the Li metal anode interface in different electrolytes, X-ray photoelectron spectroscopy (XPS) depth profiling, with the assistance of Ar ion etching, was employed to analyze the cycled electrolytes and Li anodes. In Fig. 6f, the chemical speciation of C 1*s* in p-3DSE within the solid electrolyte interface (SEI) shows minimal variation. However, there is a notable enhancement in the intensity of Li_2_O species as the sputtering time is extended. This observation serves as compelling evidence that the Li_2_CO_3_ species undergo decomposition, transitioning into the more stable Li_2_O structure at the interface between Li and p-3DSE. This phenomenon is essentially consistent across the PEGDA/LiTFSI (Fig. S30), f-SE (Fig. S31), and s-3DSE (Fig. S32). An obvious LiF peak emerges at the cycled p-3DSE and s-3DSE electrolytes, producing LiF-rich SEI (Figs. 6h and S32). In general, LiF is considered to be an important component in facilitating fast and uniform Li^+^ transportation in SEI, which would also promote uniform Li deposition [46]. In the S 2*p* and N 1*s* spectra, S 2*p* peak at 167.8 eV and N 1*s* peak at 398.6 eV corresponded to LiTFSI (Figs. 6i, k and S31, S32). The peak of ionic conductor Li_3_N at 397.4 eV in N 1*s* spectra is also considered to be an ideal component of SEI due to its ability to reduce the interfacial resistance and suppress the growth of Li dendrites [47]. In the PEGDA/LiTFSI and f-SE systems, F elements are primarily attributed to the LiTFSI component, and there is no distinct signal of LiF. As shown in Fig. 6l, there is a noticeable reduction in the intensity of C 1*s* as the sputtering time increases, accompanied by an enhancement in the intensities of F 1*s* and N 1*s*. This observation suggests the formation of an SEI film rich in LiF and Li_3_N species on the surface of p-3DSE/s-3DSE. Consequently, the establishment of a stable interface between Li metal and p-3DSE/s-3DSE is achieved, resulting in enhanced kinetics and the capability for long-term operation.

**Fig. 7:**
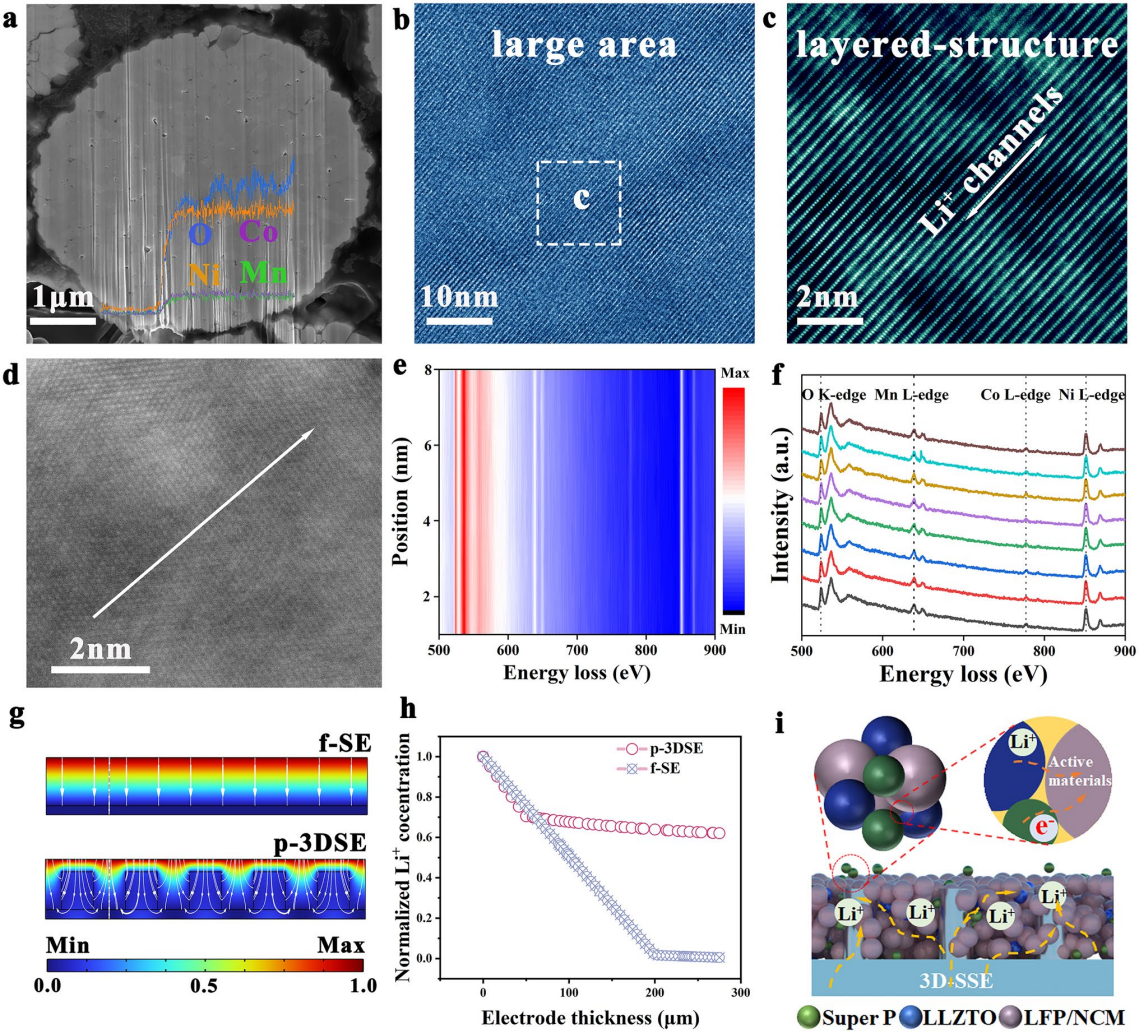
**a** Cross-sectional image of FIB-SEM and line-scan of O, Ni, Co, and Mn elements of NCM811 cathode after cycles with p-3DSE. **b**, **c** HAADF-STEM images of the interior region for FIB-prepared NCM811. **d**–**f** High-resolution STEM image, and the corresponding EELS spectra of Co, Ni, Mn L-edge, and O K-edge. **g** Li^+^ concentration distribution of the integrated electrolyte/cathode at 200 μm for f-SE and p-3DSE electrolytes, and **h** the corresponding relationship between Li^+^ concentration and electrode thickness along the vertical direction (the line in Fig. 7 g). **i** Schematic illustration of the Li^+^ transport mechanism between p-3DSE and cathode materials

The positive effect of the p-3DSE electrolyte on the NCM811 and LFP cathodes in terms of thier rate and long-term electrochemical performance was pronounced in coin full cells featuring with Li metal anode. The cross-sectional SEM image of the cycled NCM811 cathode with p-3DSE electrolyte showed their microstructural characteristics in the mechanical stability. As shown in Fig. 7a, the secondary particles of a cycled NCM811 cathode contain no visible microcracks, while those of a cycled NCM811 cathode with Poly(PEGDA)/LiTFSI electrolyte are nearly fractured and contained extensive networks of wide microcracks (Fig. S33). SEM–EDS analysis revealed the uniform distribution of O, Ni, Co and Mn elements from the surface to the bulk of cycled NCM811 particles. Furthermore, the crystal structure of NCM811 cathode after 300 cycles at 0.2C was investigated by the atomic resolution Z-contrast STEM-HAADF imaging. As shown in Fig. S34, the NCM811 cathode with Poly(PEGDA)/LiTFSI electrolyte is accompanied by a large amount of cation mixing and NiO phase near the crack, which greatly inhibited the transport of Li^+^ and rapidly increased the electrochemical impedance (Fig. S35). On the contrary, the layered structure with R-3 m space group was well retained in the bulk of cycled NCM811 cathode with p-3DSE electrolyte (Fig. 7b, c), and the stable layered structure can provide a stable diffusion channel for Li^+^ in the electrochemical performance, which demonstrated fast kinetics and electrochemical cycle stability, which can be demonstrated by CV curves using different electrolytes (Fig. S36). Electron energy loss spectroscopy (EELS) spectra were tested to reveal the electronic structure evolution of Ni, Co, Mn and O elements. The line in Fig. 7d indicates the measurement position of EELS. The chemical shift of the Ni L-edges, Co L-edges, and Mn L-edges of the NCM811 cathode with Poly(PEGDA)/LiTFSI electrolyte to the position with low energy loss, especially the Ni, indicated the reduction of the valence state of the metal element. In addition, the O K-edges strength decreases gradually from the bulk to the surface, indicating the formation of oxygen vacancy and promoting the migration of transition metal ions to the Li^+^ sites, exacerbating the structural transformation (Fig. S37). However, the Ni L-edges, Co L-edges, Mn L-edges, and O K-edges of NCM811 cathode with p-3DSE electrolyte show almost no chemical shift, which is indicative of the structure stability. All these findings contribute to the enhanced compatibility of p-3DSE electrolytes with the high-voltage cathode (NCM811). Such superior full cells performances can be ascribed to the high oxidation stability of p-3DSE due to the addition of LLZTO inorganic networks, the high Li^+^ conductivity at room temperature and the intimate contact between Li metal anode and p-3DSE (Fig. 7i).

To elucidate the origin of the enhanced interfacial Li^+^ transportation by the introduction of vertical-aligned ion-conducting network on cathodes, Multiphysics simulation were performed to analyze the ion distribution (Fig. S38). The Li^+^ concentration distribution under two cathode/electrolyte models were simulated, as shown in Fig. 7g. The results show that a more homogeneous Li^+^ distribution and negligible Li^+^ concentration polarization in the p-3DSE/cathode are achieved compared with that of the Poly(PEGDA)/LiTFSI/cathode. From the f-SE/cathode model, large concentration gradients are observed due to the long transport pathway of Li^+^ which is unfavorable for efficient Li^+^ migration and diffusion, especially under high-load electrodes. The p-3DSE case with micro-pillars provides a facile pathway for Li^+^ transport which minimize the transport length resulting in enhanced Li^+^ transport performance. Therefore, these achievements result in a low concentration gradient, and hence low concentration polarization, which leads to the optimized rates performance. The relationship between Li^+^ concentration and electrode thickness along the vertical direction is shown in Fig. 7h. The local Li^+^ concentration is larger in the p-3DSE/cathode electrode with the increasing thickness of integrated electrodes than that of the f-SE/cathode electrode when the electrode thickness is larger than 62 μm. This indicates a better Li^+^ transfer and lower Li^+^ diffusion resistance for the p-3DSE/cathode electrode. Thus, high capacity can be achieved by loading cathode materials on the as-designed 3D electrolyte with pillared-structure arrays. This structure enables a continuous Li^+^ conduction network, which demonstrates rapid and stable Li^+^ transfer within the electrolyte/cathode (Fig. 7i).

## Conclusion

In summary, 3D composite solid electrolytes with highly efficient ion-conducting networks are developed using 3D printing technologies. Multiple-type electrolyte films with vertical-aligned micro-pillar (p-3DSE) and spiral (s-3DSE) structures were rationally designed and fabricated. The results demonstrates that p-3DSE homogenized the Li^+^ ion concentration at the electrolyte/Li interfaces, reinforce the electrolyte/cathode interfacial adhesion, and improve the loading of cathode materials. The 3D-printed p-3DSE delivered robust long-term cycle life of up to 2600 cycles at 1 mA cm^−2^ and a high critical current density of 1.92 mA cm^−2^. The optimized 3D electrolyte structure could realize all-solid-state Li metal batteries with a dramatically superior full-cell areal capacity of 2.75 mAh cm^−2^ (LFP) and 3.92 mAh cm^−2^ (NCM811) at the room temperature. The novel design of 3D-printed electrolytes showed excellent interfacial stability with Li anode and LFP (NCM811) cathode, thus preventing interfacial degradation induced by the dendrite growth and the contact loss. Our study describes a highly efficient Li^+^ transport mode of CSEs for advanced solid-state lithium metal batteries.

## Supplementary Information

Below is the link to the electronic supplementary material.

Supplementary file1 (PDF 2732 kb)
