# Supplementary material for: Highly Efficient Aligned Ion-Conducting Network and Interface Chemistries for Depolarized All-Solid-State Lithium Metal Batteries
Source: Nanomicro Lett. 2024 Jan 12;16:86. doi: 10.1007/s40820-023-01301-4 (PMC10786779; doi:10.1007/s40820-023-01301-4)
Supplement: Supplementary file 1 — Supplementary file1 (PDF 2732 kb) [file 40820_2023_1301_MOESM1_ESM.pdf]

Supporting Information for

Highly Efficient Aligned Ion-Conducting Network and Interface Chemistries for Depolarized All-Solid-State Lithium Metal Batteries

Yongbiao Mu[1,2,3,‡], Shixiang Yu[1,2,3,‡], Yuzhu Chen [1,‡],Youqi Chu[1,2,3,‡], Buke Wu[1,2,3], Qing Zhang[1,2,3], Binbin Guo[1], Lingfeng Zou [1,2,3], Ruijie Zhang[1,2,3], Fenghua Yu[1,2,3], Meisheng Han[1,2,3], Meng Lin[1,*], Jinglei Yang [1,2,3,*], Jiaming Bai[1,*], Lin Zeng[1,2,3,*]

[1] Shenzhen Key Laboratory of Advanced Energy Storage, Southern University of Science and Technology, Shenzhen 518055, P. R. China

[2] Department of Mechanical and Energy Engineering, Southern University of Science and Technology, Shenzhen 518055, P. R. China

[3] SUSTech Energy Institute for Carbon Neutrality, Southern University of Science and Technology, Shenzhen 518055, P. R. China

[4] Department of Mechanical and Aerospace Engineering, Hong Kong University of Science and Technology, 997077, Hong Kong Special Administrative Region of China

[5] HKUST Shenzhen-Hong Kong Collaborative Innovation Research Institute, Futian, Shenzhen, P. R. China

[‡] Yongbiao Mu, Shixiang Yu, Yuzhu Chen, and Youqi Chu contributed equally to this work.

*Corresponding authors. E-mail: linm@sustech.edu.cn (M. Lin); maeyang@ust.hk (J. Yang); baijm@sustech.edu.cn (J. Bai); zengl3@sustech.edu.cn (L. Zeng)

S1 Characterizations

The morphologies were characterized by Hitachi SU-8230 field emission scanning electron microscopy (SEM, Hitachi SU-8230). XRD (New D8 Advance, Bruker) was employed to characterize the crystal structure of LLZTO using Cu Kα radiation in the 2θ range of 10°–80° with a step size of 0.2°. Transmission electron microscopy (TEM), energy-dispersive X-ray analysis (EDX) and elemental mapping were performed using a Talos instrument with an acceleration voltage of 300 kV. Thermogravimetric analysis (TGA) was performed with a PerkinElmer TGA4000 thermogravimetric analyzer from 30 to 1000 ℃ with a ramping rate of 5 ℃ min^{-1} under air. X-ray photoelectron spectra (XPS, Escalab 250Xi) were acquired on a Thermo SCIENTIFIC ESCALAB 250Xi with Al Kα (hυ = 1486.8 eV) as the excitation source. Raman spectra were performed on a HORIBA LabRAM HR Evolution using a 532 nm laser as the excitation source. The micro-CT images were detected by the Diondo D2 scan system. Fourier transform infrared spectroscopy (FT-IR) spectra were performed in a Thermo Nicolet AVATAR

360 from 4000 to 500 cm^{-1}. To observe the morphologies evolution of the cycled Li metal and electrolytes at high resolution, a FIB-SEM was used to prepare and analyze the samples. To characterize the chemical species of the SEI film, XPS data at different depths were performed via a transfer chamber to prevent contact of the sample with moisture and air. The C 1s peak at 284.8 eV corresponding to C-C bonding was used as a reference for charge compensation.

Testing procedure of the stress-strain curves: The testing procedure involved the sample preparation, initial configuration, stress-strain testing, and data analysis for PEO/LiTFSI, f-SE, p-3DSE, and s-3DSE. Sample preparation involves creating uniform membrane samples for each material with consistent dimensions and thickness. These samples were securely mounted onto a mechanical testing machine's grips to ensure a secure attachment without inducing stress concentrations. The initial configuration set the sample's gauge length and zero applied load. The testing machine was calibrated to ensure accurate force and displacement measurements, and tests were conducted under controlled environmental conditions, typically at room temperature. The stress-strain testing began by applying a controlled tensile load to the sample at a constant rate, typically between 1 and 10 mm/min. Simultaneously, the testing machine's sensors recorded the applied force (stress) and the corresponding deformation (strain). Testing continued until one of the following criteria was met: the sample fractures, the desired strain limit was reached, or specific study-related parameters or conditions were satisfied. Continuous data collection during the test generated a stress-strain curve for each material, illustrating how each material responds to mechanical deformation. Subsequent data analysis extracted mechanical properties, including Young's Modulus (elasticity), Ultimate Tensile Strength (maximum stress before failure), Elongation at Break (strain at which the sample fractures), and other relevant parameters. This comprehensive testing procedure offered critical insights into the mechanical behavior and properties of the membrane materials under tensile loading conditions.

S2 Electrochemical Measurements, Calculations, and Simulations

Battery Assembly: For symmetric cell, galvanostatic cycling testings under different current densities were conducted to investigate the cyclability of Li/3DSE/Li and Li/SE/Li symmetric cells. Li/3DSE/Li cells were assembled by sandwiching the 3DSE between two Li-metal chips with a thickness of 500 μm. The electrochemical tests of symmetric cells were performed from 25 °C (room temperature) to 100 °C. For all-solid-state Li metal full-cell fabrication, LFP/3DSE and NCM811/3DSE composite cathodes were first prepared by the spraying method. For a comparison, LFP and NCM811 cathodes were also prepared by the conventional slurry casting method. 70 wt% LFP or NCM811 powders, 10 wt% inorganic ceramic powder $Li_{6.5}La_3Zr_{1.5}Ta_{0.5}O_{12}$ (LLZTO), 10 wt% carbon black (CB), and 10 wt% polyvinylidene fluoride (PVDF) were mixed in N-methyl-2-pyrrolidinone, and then the slurry was cast on Al foil to form

a composite cathode after drying for 3 h in drying oven at 60 °C, then 24 h in a vacuum oven at 100 °C. The cathodes had an active material mass loading of about 2 mg cm^{-2}. Ultra-thin Li foil (50 μm) was purchased from China Energy Lithium Co., Ltd.. LIR2032 type coin cell or pouch cell were assembled with the above cathodes, Li anode, and various electrolyte films in the Ar-filled glove box.

Electrochemical Measurements: For ionic conductivity measurements, Poly(PEGDA)/LiTFSI, s-3DSE and p-3DSE polymer composite electrolytes were assembled into a coin-cell with stainless steel (SS) blocking electrodes. Electrochemical impedance spectroscopy (EIS) was performed in the temperature range of 20-100 °C using a CHI 660 electrochemical workstation in the frequency range of 1 MHz to 0.1 Hz. The ionic conductivity (σ) was given by Eq. (S1):

$$\sigma = L/RS \tag{S1}$$

where R is the resistance value of the bulk solid electrolyte; L is the thickness of the electrolyte membrane (~ 275 μm), and S is the effective area of contact between the electrolyte and the stainless-steel blocking electrode (1.13 cm^2).

Electrochemical Voltage Window: Linear sweep voltammetry (LSV) was conducted on both pure Poly(PEGDA) and LLTO framework composite electrolytes to see the electrochemical voltage window. Attached with a lithium metal foil on one side and a stainless steel as the blocking electrode on the other side, LSV was conducted from 0 to 6 V at room temperature. The scan rate was fixed to 1.0 mV sec^{-1}.

Lithium Ion Transference Number: In the t_{Li^+} measurements, Li/SE/Li, Li/s-3DSE/Li, Li/p-3DSE/Li symmetric cells were assembled to test the potentiostatic direct current (DC) polarization and EIS measurements. The lithium-ion transference number (t_{Li^+}) was calculated using Eq. (S2):

$$t_{Li^+} = \frac{I_s(\triangle V - I_0 r R_1^0)}{I_0(\triangle V - I_s R_1^s)} \tag{S2}$$

where ΔV is the applied DC voltage (10 mV); I_0 and I_s are the initial and steady currents, respectively, and R_1^0 and R_1^s are the charge-transfer resistances before and after DC polarization, respectively.

Li ions distribution simulation

Governing equations: The ion distribution profiles are obtained by performing fintite element based simultions for various geometry designs (Table S5). The transport of Li ions in solid electrolytes, i.e., diffusion and electro-migration, are described by the Nernst-Planck formulation. The governing equations, considering mass balance, species transport, electro-neutrality, and current conservation, are given as:

$$J_i = -D_i \nabla c_i - z_i u_i F c_i \nabla \phi_l \qquad \text{(S3)}$$

$$\nabla \cdot J_i = R_i \qquad \text{(S4)}$$

$$\sum_i z_i c_i = 0 \qquad \text{(S5)}$$

$$\nabla \cdot i_l = F \sum_i z_i R_i \qquad \text{(S6)}$$

where J_i is the mass flux, D_i is the diffusion coefficient, c_i is the concentration, z_i is the charge number, u_i is the mobility (defined by the Nernst-Einstein equation), ϕ_l is the electrolyte potential. $R_i = \frac{-v_i i_{loc}}{2F}$ is the electrochemical reaction source term (i_{loc} is the local current density at the electrode surface, v_i is the stoichiometric coefficient, and F is the Faraday constant). $i_l = \sum_m i_{loc,m}$ is the current density of electrolyte (m indicates the charge transfer electrode reaction).

Andoe electrode model with different types of electrolytes: The 2D geometrical dimensions and major boundary conditions are shown in Fig. S27. The Li^+ diffusion coefficients of different types of electrolytes are 3.2×10^{-12}, 1.6×10^{-11}, 6.6×10^{-10} cm^2/s for planar, spiral, and pillar model, respectively. The domain of Li anode is in rectangle shape with a length (D_{Li}) of 1.4 mm and a thickness (H_{Li}) of 450 µm. The thickness (H_{planar}) of planar electrolyte is 37.5 µm, and the length of planar electrolyte is the same as that of Li anode. The domain of spiral electrolytes includes a substrate and four pillars with different spacing. The thickness (H_{sub}) of substrate is 37.5 µm and the length of substrate is also the same as that of Li anode. The thickness (H_{spiral}) and length (D_{spiral}) of the four pillars are 150 µm and 100 µm. The distance ($D_{spiral,in}$) between the centers of the two inner pillars is 300 µm and the distance ($D_{spiral,out}$) between the centers of the two outer pillars is 400 µm. The domain of pillar electrolyte includes a substrate and three pillars with a same spacing of 400 µm. The dimension of the substrate in the pillar electrolyte model is the same as that in the spiral electrolyte. The distance ($D_{pillar,inter}$) between the centers of the pillars is 400 µm. Boundary conditions for three models were shown in **Fig. S29**. The initial concentration of Li^+ and the magnitude of the external electric current density were set at the top of solid electrolyte (yellow lines). The concentration of Li^+ was set to be 1 M, and the external electric current density flows into electrolyte was set to be −0.3 mA/cm^2. The surface of Li anodes has a boundary electric potential of 0 V (red lines). The rest boundaries are considered as insulation.

Cathode electrode model: The geometrical dimensions and major boundary conditions of the two physical models, i.e., f-SE and p-3DSE were shown in **Fig. S38**. Note that for both cases a same thickness of cathode electrode are assumued. The Li^+ diffusion

coefficient ($D_{cathode}$) of cathode electrode for planar and p-3DSE electrolyte model is assumed to be 5.0 × 10^{-13} cm^2 s^{-1}. The p-3DSE electrolyte model contains a substrate with five pillars. The thickness (H_{sub}) and length of the substrate (D_L) are 37.5 µm and 2 mm, respectively (the dimension of substrate in p-3DSE electrolyte model was the same as that of p-3DSE electrolyte model). The thickness (H_{pillar}) and length (D_{pillar}) of pillar were 150 µm and 100 µm. The thickness of cathode electrode in planar and p-3DSE electrolyte model equals 200 µm. The initial concentration of Li^+ and the magnitude of the external electric current density were set at the top of solid electrolyte (yellow lines). The concentration of Li^+ was set to be 1 M, and the external electric current density flows into the electrolyte was set to be 0.3 mA cm^{-2}. The surface of Li anodes has a boundary electric potential of 0 V (red lines). The rest boundaries are insulation.

Numerical methods: All models were solved in commercial software, COMSOL Multiphysics, using the finite element method. The grid-independence study was performed with an increasing number of mesh element numbers from 3.1 × 10^4 to 3.2 × 10^5. The mesh numbers of computation domains for were discretized into 3.6 × 10^4, 6.8 × 10^4, and 9 × 10^4 elements for planar SE, s-3DSE, and p-3DSE electrolyte model, respectively. The mesh numbers of computation domains were discretized into 4.8 × 10^4, and 6.5 × 10^4 elements for f-SE and P-3DSE models, respectively. The convergence was dictated to ensure the variation of mean Li^+ concentration at the electrolyte-Li anode interfaces less than 0.01% as a further increase of mesh numbers. A fully coupled Newton method, using a PARDISO solver, with a relative error of 1 × 10^{-5} was used to solve all coupled PDEs involved.

S3 Supplementary Figures and Tables

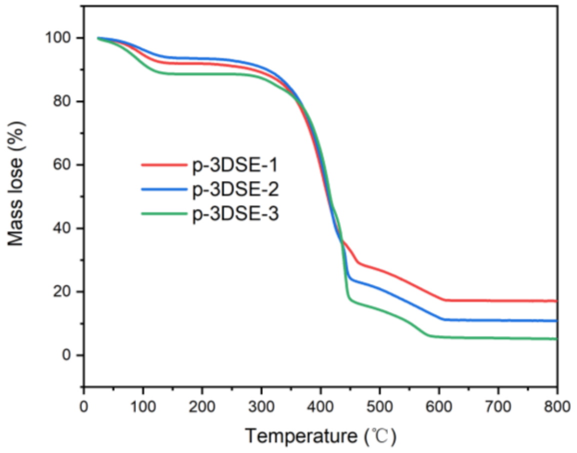

Fig. S1 TGA curves of various p-3DSE varying the amount of nanoscale LLZTO

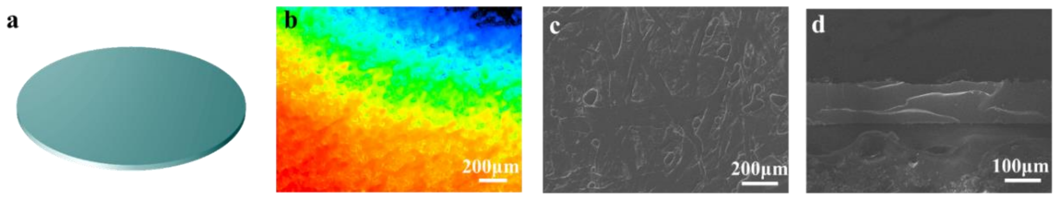

Fig. S2 a The structure diagram of planar structure electrolyte (2DSE). **b** LCSM image of 2DSE and corresponding SEM images of **c** top-view and **d** cross-sectional view

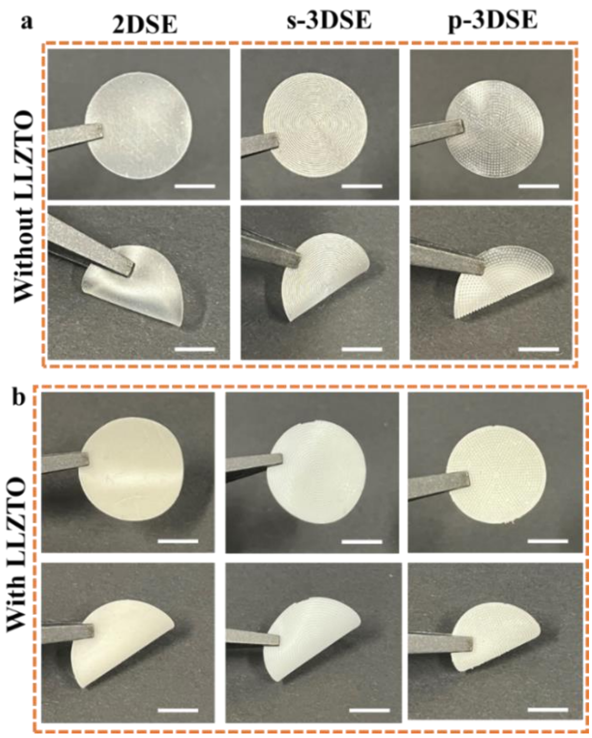

Fig. S3 The optical images of 3D-printed 2DSE (f-SE), s-3DSE and p-3DSE of **a** without LLZTO and **b** with LLZTO inorganic electrolyte (The diameter of the circular electrolyte is 16mm)

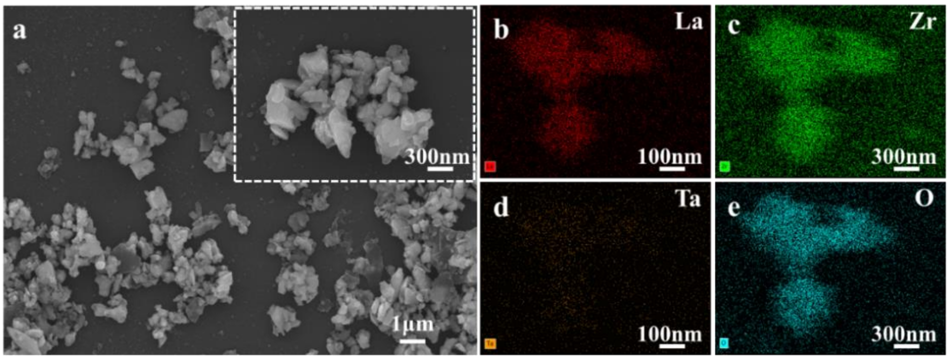

Fig. S4 a SEM images of nanoscale LLZTO inorganic electrolyte powder prepared by high-energy ball mill method, and corresponding elements mapping of **b** La, **c** Zr, **d** Ta, and **e** O

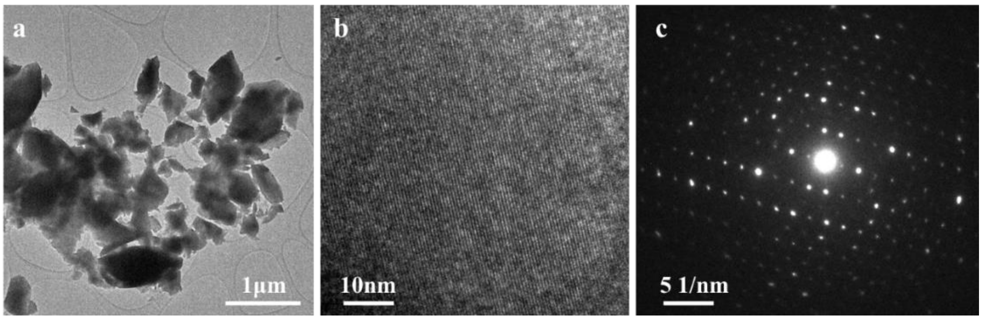

Fig. S5 a TEM images of nanoscale LLZTO inorganic electrolyte powder prepared by high-energy ball mill method, **b** corresponding HRTEM image and **c** SAED image

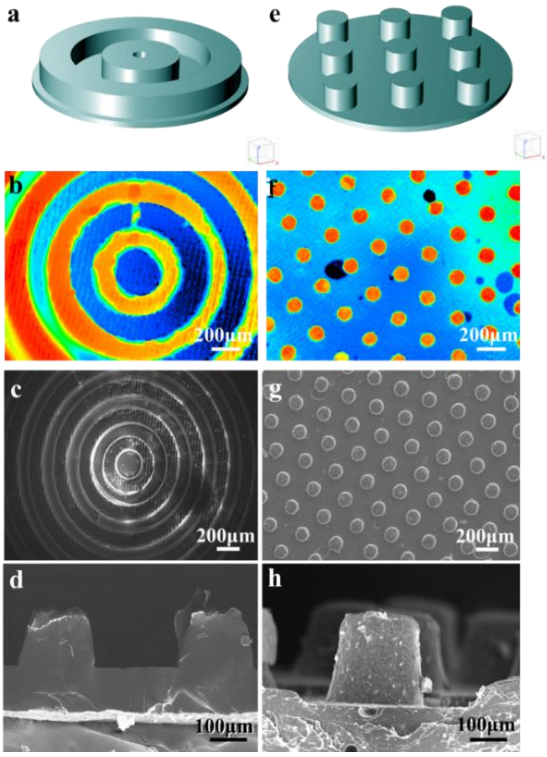

Fig. S6 The structure diagram of **a** s-3DSE electrolyte and **e** p-3DSE electrolyte. **b, f** the corresponding LCSM images and SEM images of **c, g** top-view and **d, h** cross-sectional view for s-3DSE electrolyte and p-3DSE electrolyte, respectively

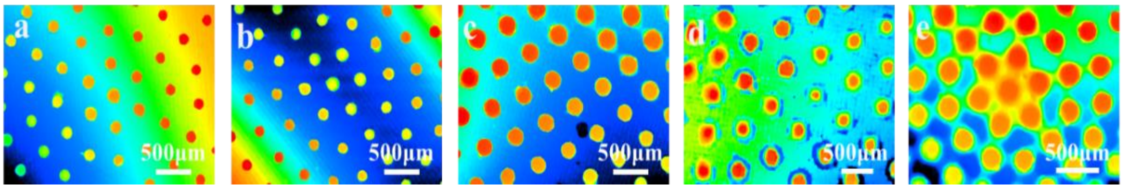

Fig. S7 2D laser scanning confocal microscopy (LSCM) images of p-3DSE electrolytes with different sizes in terms of the diameter and density of the pillars

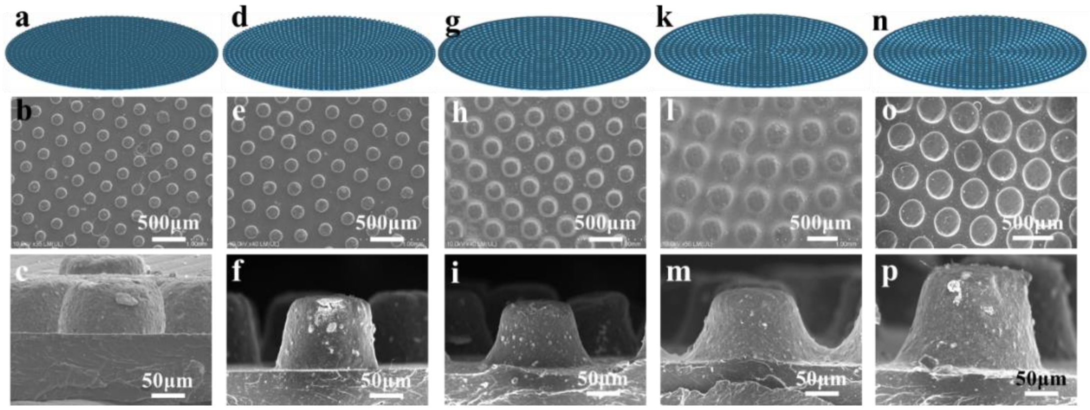

Fig. S8 a, d, g, k, n The structure diagram of different p-3DSE electrolyte. The corresponding SEM images of **b, e, h, l, o** top-view and **c, f, i, m, p** cross-sectional view for p-3DSE electrolyte, respectively

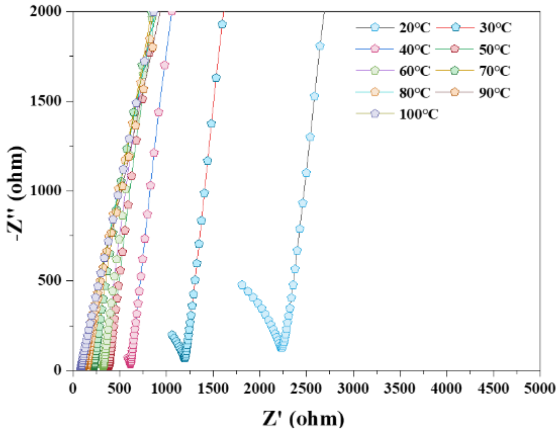

Fig. S9 The Nyquist plot of the SS/s-3DSE/SS cell in a temperature range from 20 to 100 °C

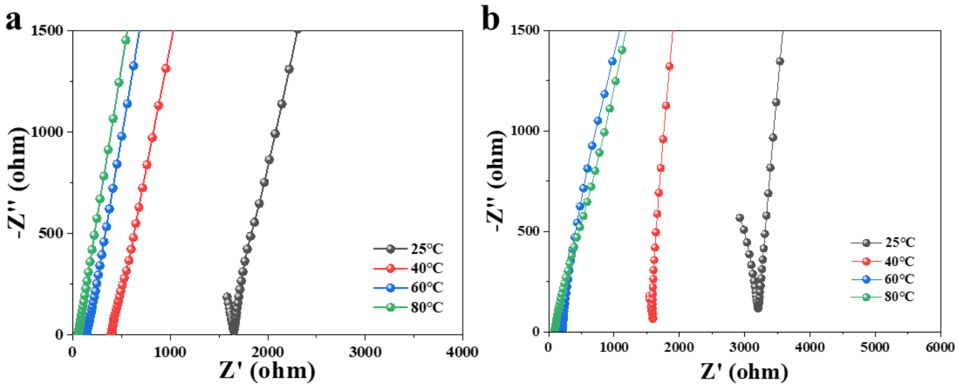

Fig. S10 The Nyquist plot of **a** the SS/f-SE/SS and **b** SS/Poly(PEGDA)/LiTFSI/SS cells in a temperature range from 25 to 80 °C

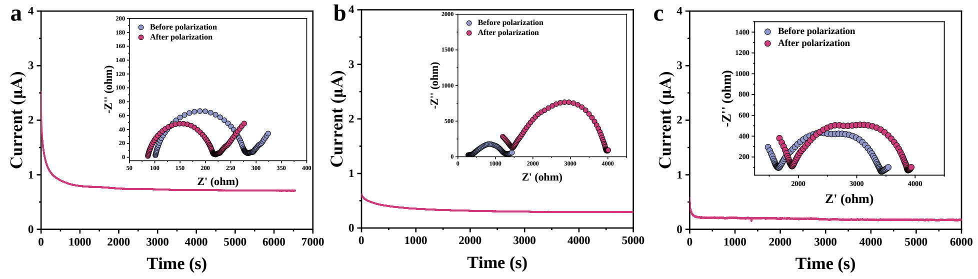

Fig. S11 The t_{Li+} of **a** s-3DSE, **b** f-SE, and **c** Poly(PEGDA)-LiTFSI electrolytes

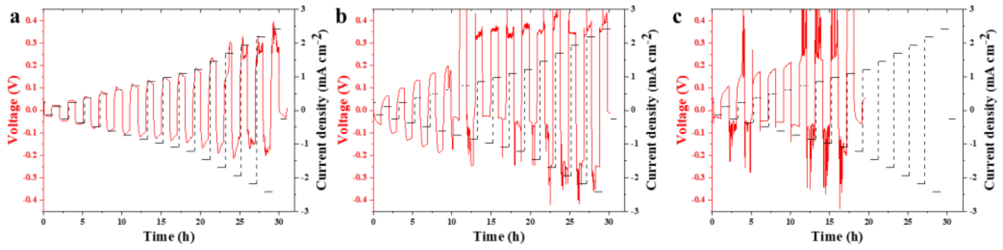

Fig. S12 The critical current density (CCD) for **a** s-3DSE, **b** f-SE and **c** Poly(PEGDA)-LiTFSI electrolytes

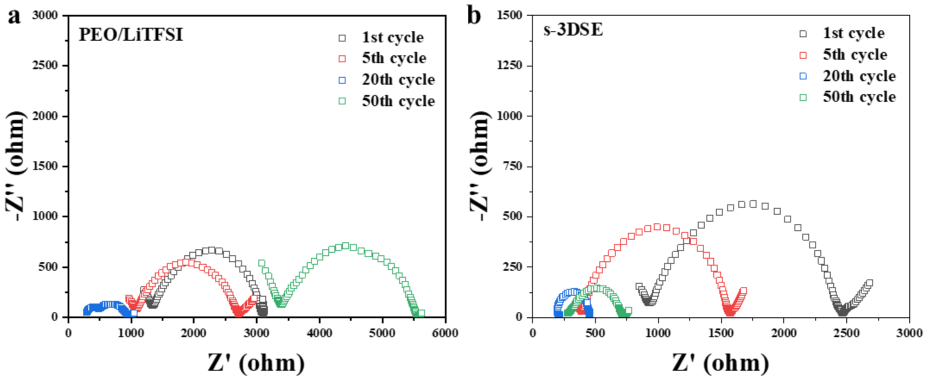

Fig. S13 EIS spectra of the **a** Li/ Poly(PEGDA)/LiTFSI/Li and **b** Li/s-3DSE/Li cells cycled after different cycles

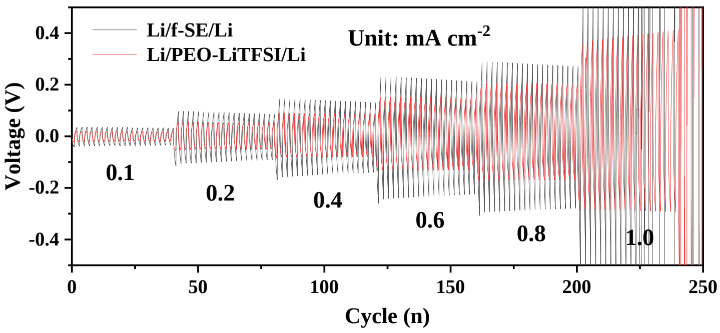

Fig. S14 The rate performance of **a** Li/ Poly(PEGDA)/LiTFSI/Li cells and **b** Li/f-SE/Li cells

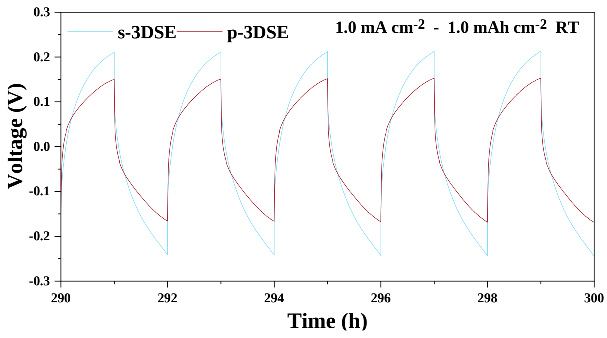

Fig. S15 The corresponding enlarged view of the voltage profiles in 290–300 h

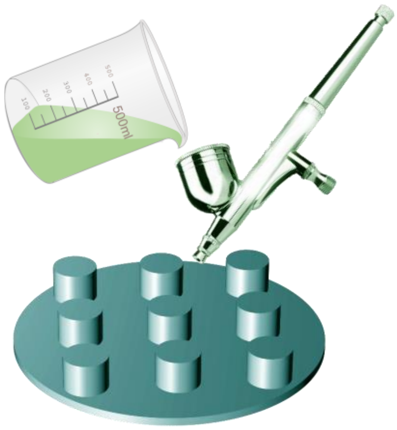

Fig. S16 Schematic showing the fabrication process of various p-3DSE/LFP and p-3DSE/NCM811 cathodes by a simple spraying process

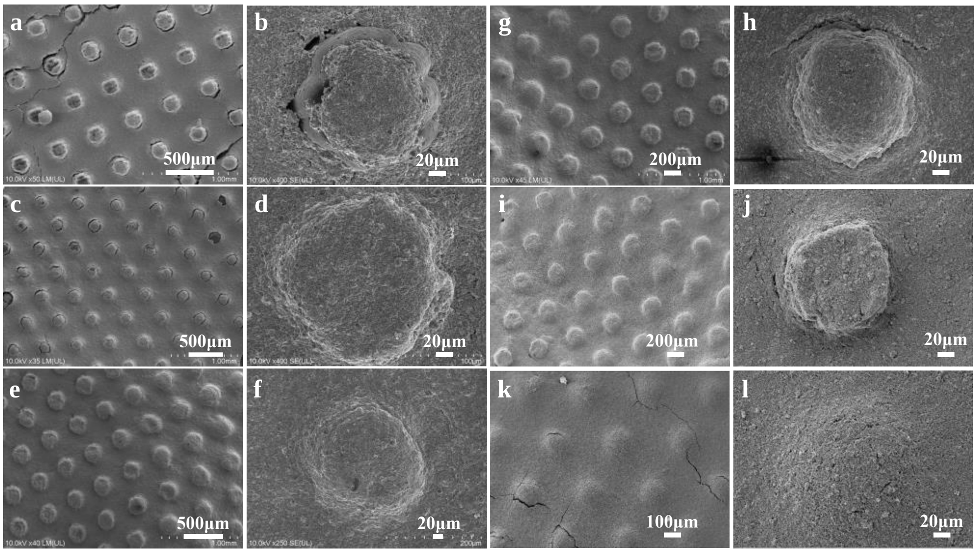

Fig. S17 SEM images of various p-3DSE/LFP cathodes by a spraying method, and the corresponding mass loading of **a, b** 3.5 mg cm^{-2}, **c, d** 8.5 mg cm^{-2}, **e, f** 10.0 mg cm^{-2}, **g, h** 12.7 mg cm^{-2}, **i, j** 15.5 mg cm^{-2}, **k, l** 20.0 mg cm^{-2}

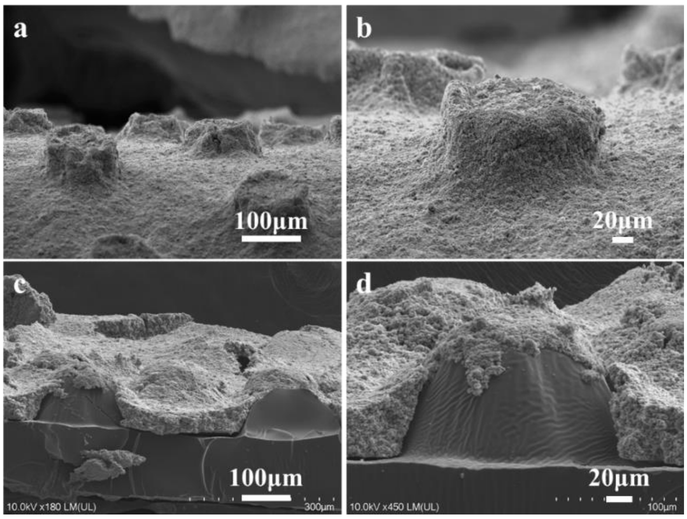

Fig. S18 Cross-sectional SEM images of p-3DSE/LFP cathodes **a, b** by a spraying method and **c, d** traditional method

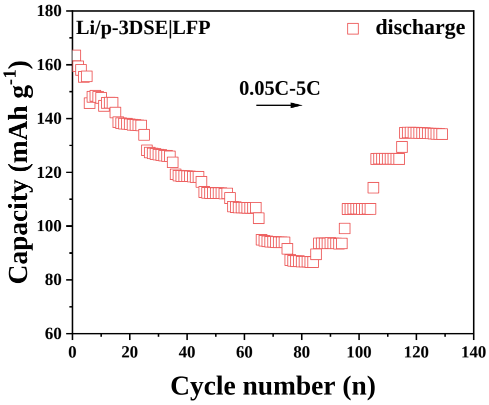

Fig. S19 Rate performance of Li/p-3DSE/LFP full cell at various rates from 0.05 C to 5 C (1C=172 mAh g^{-1})

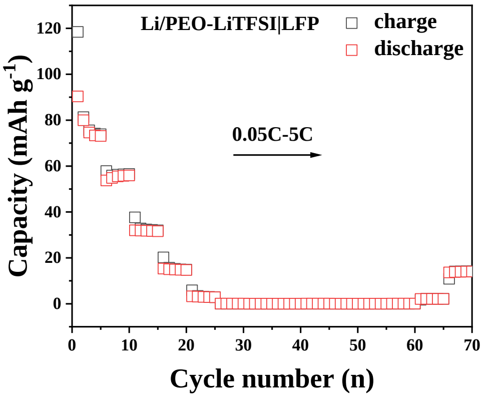

Fig. S20 Rate performance of Li/ Poly(PEGDA)-LiTFSI/LFP full cell at various rates from 0.05 C to 5 C (1C = 172 mAh g^{-1})

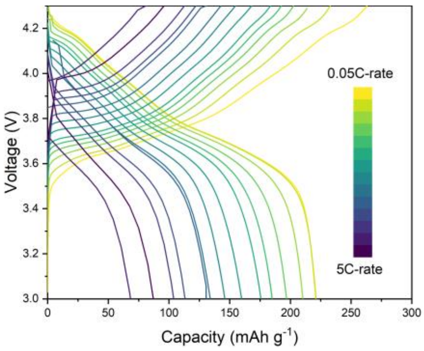

Fig. S21 CV curves of various Li/p-3DSE/NCM full-cells at 0.1 mV s^{-1} from 2.7 V to 4.3

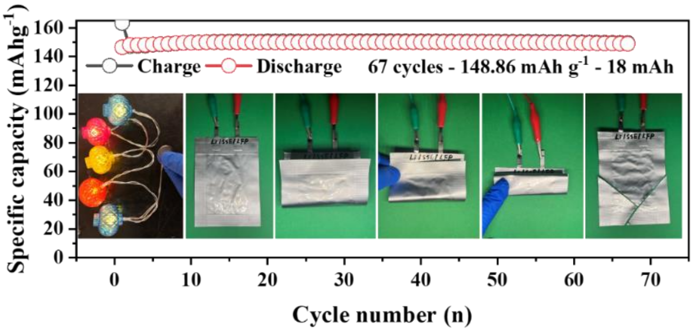

Fig. S22 Cycle performance of all-solid-state Li/p-3DSE/LFP full cell with a capacity of 18 mAh at 0.1 C under different bending states. (Insets are the optical images of pouch cells)

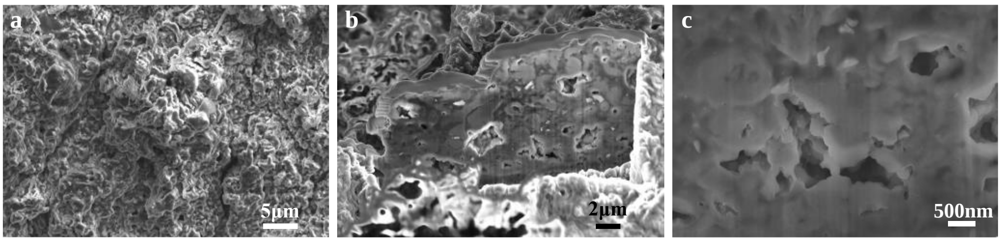

Fig. S23 a SEM image of a Li metal cycled beyond 1000 times under Poly(PEGDA)/LiTFSI electrolyte **b** SEM image of a after FIB cutting; **c** enlarged SEM image of (b) in BSE mode

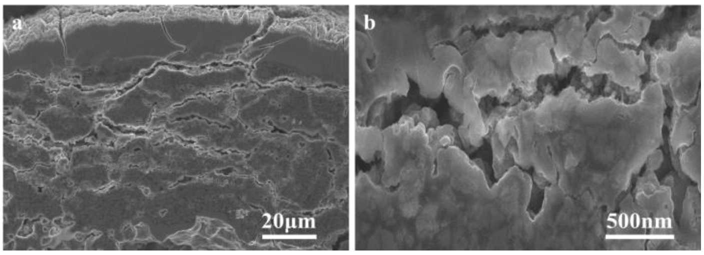

Fig. S24 a SEM image of Poly(PEGDA)/LiTFSI electrolyte after repeated cycles, **b** enlarged SEM image of (a)

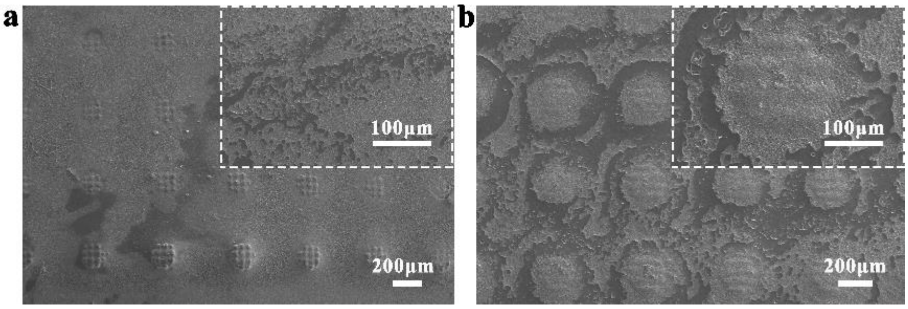

Fig. S25 The plating process of Li metal on the p-3DSE. SEM images of p-3DSE at different stages of **a** 2th cycle and **b** 10th cycle (Insets are enlarged SEM images)

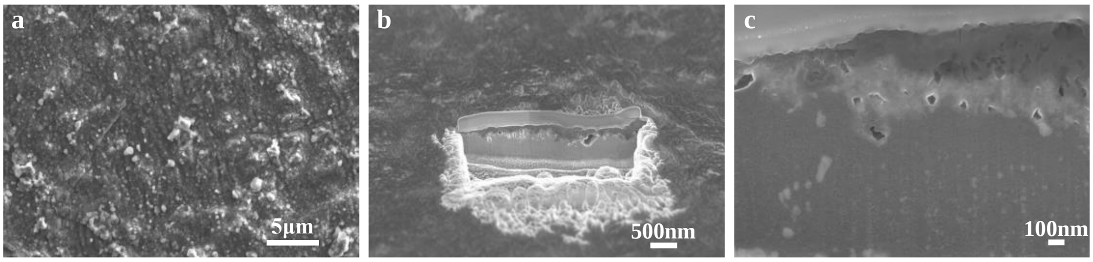

Fig. S26 a SEM image of a Li metal cycled beyond 1000 times under p-3SDSE electrolyte **b** SEM image of (a) after FIB cutting in BSE mode

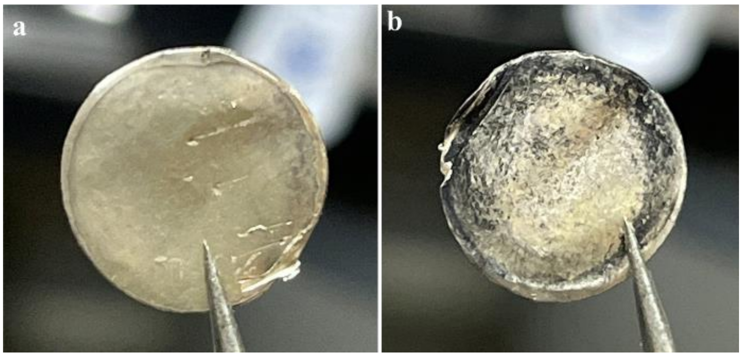

Fig. S27 The optical images of Li metal after cycles with **a** p-3DSE electrolyte and **b** Poly(PEGDA)/LiTFSI electrolyte (the diameter of Li metal is 12mm)

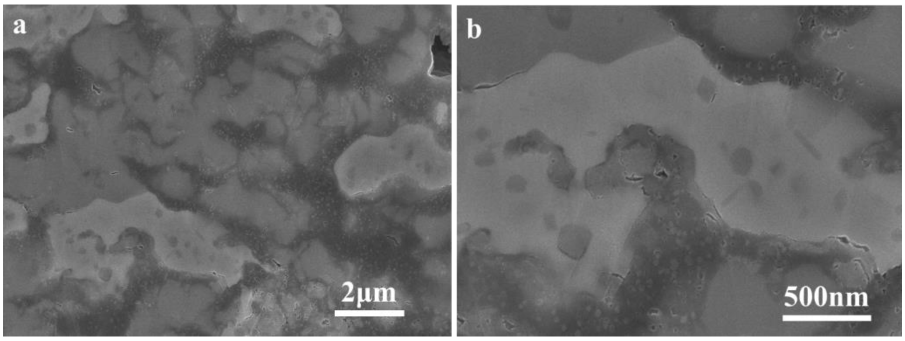

Fig. S28 a SEM image of p-3DSE electrolyte after repeated cycles, **b** enlarged SEM image of (a) from cross-sectional view after FIB cutting

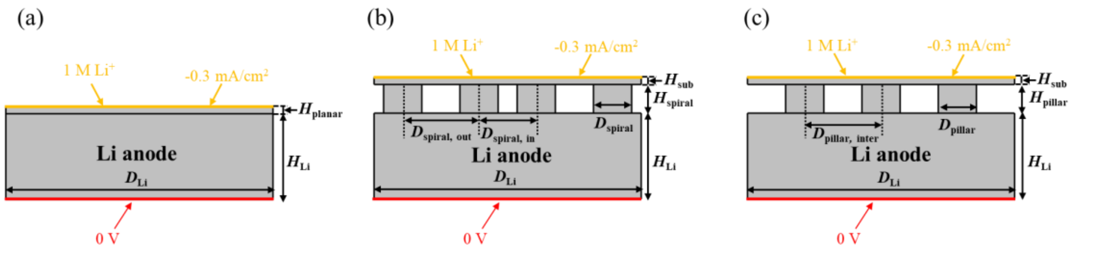

Fig. S29 The geometrical structures and boundary conditions for **a** planar electrolyte; **b** s-3DSE electrolyte; **c** p-3DSE electrolyte. Yellow lines indicate the boundary conditions of initial Li^+ concentration and inflow electric current density. Red lines indicate a boundary electric potential of 0 V

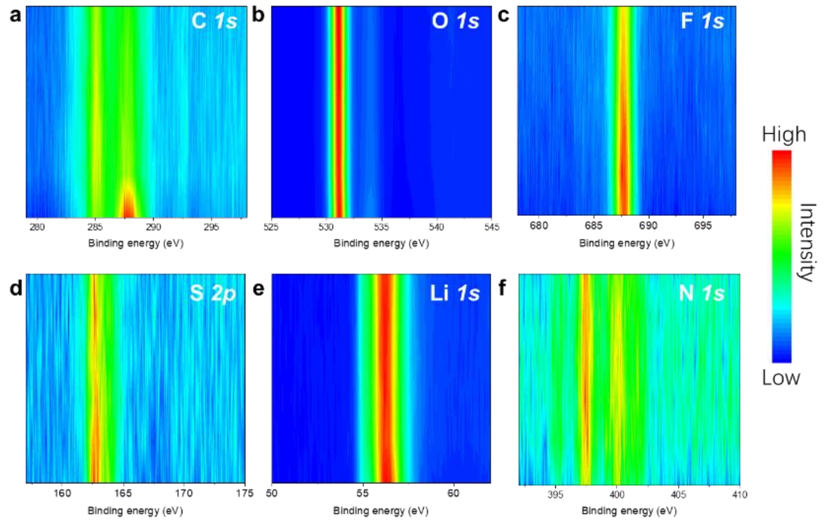

Fig. S30 XPS depth curves with the PEGDA/LiTFSI electrolyte

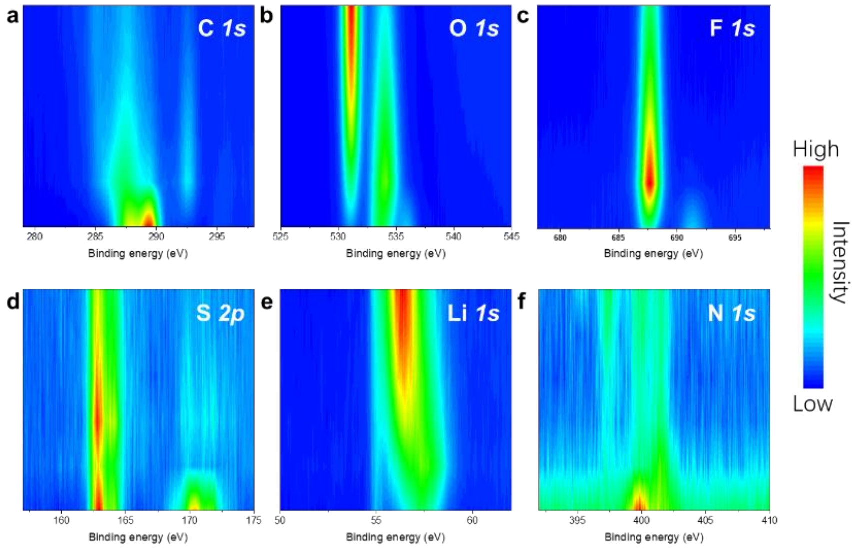

Fig. S31 XPS depth curves with the f-SE electrolyte

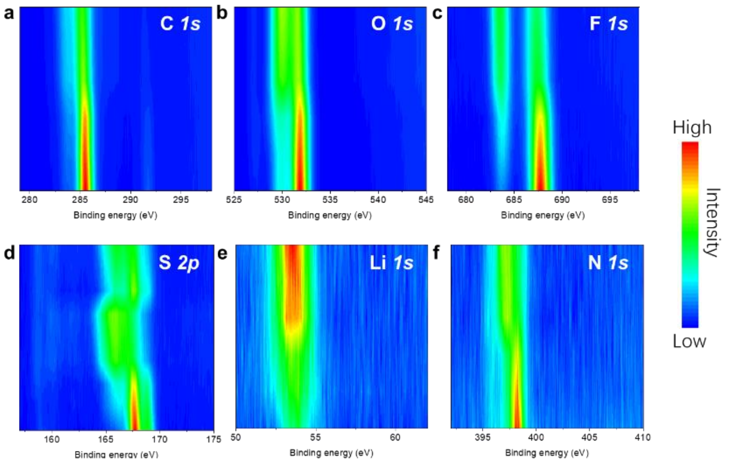

Fig. S32 XPS depth curves with the s-3DSE electrolyte

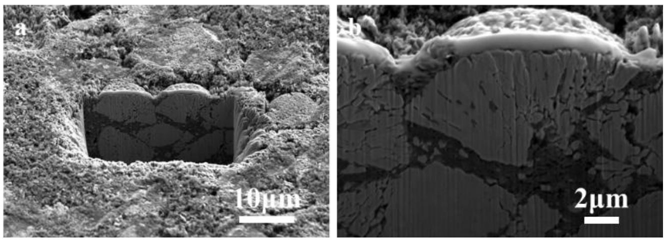

Fig. S33 The cross-sectional SEM image of the cycled NCM811 cathode with Poly(PEGDA)/LiTFSI electrolyte

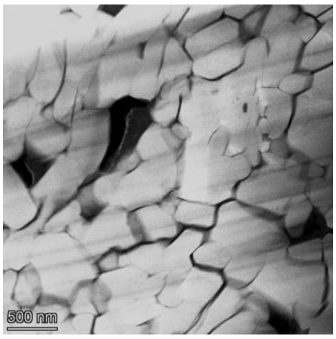

Fig. S34 The atomic resolution Z-contrast STEM-HAADF image prepared by FIB

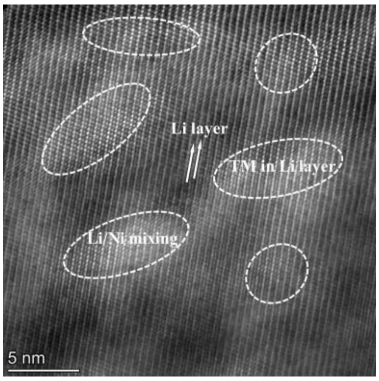

Fig. S35 HAADF-STEM images of the interior region for FIB-prepared NCM811 cathode after cycles with Poly(PEGDA)/LiTFSI electrolyte

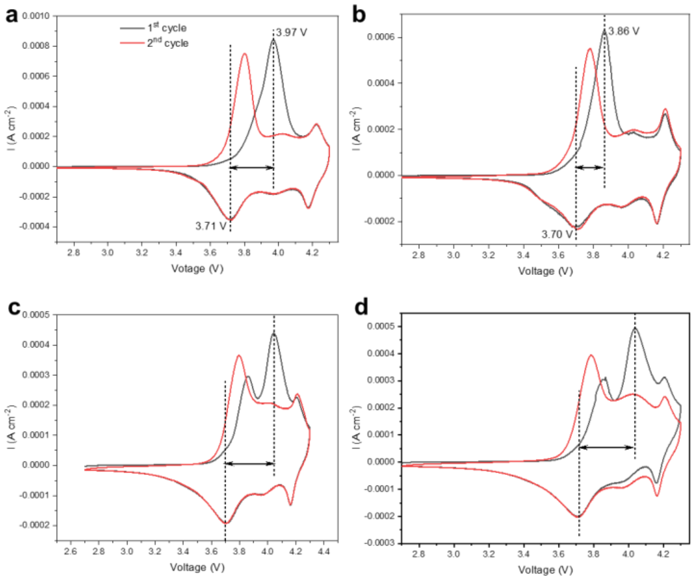

Fig. S36 CV curves of various Li/electrolyte/NCM full-cells at 0.1 mV s^{-1} from 2.7 V to 4.3 V: **a** Li/s-3DSE/NCM; **b** Li/p-3DSE/NCM; **c** Li/f-SE/NCM; and **d** Li/PEGDA/LiTFSI/NCM

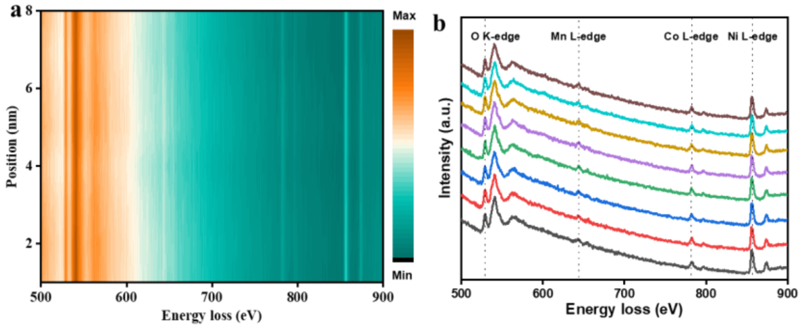

Fig. S37 EELS spectra of Co, Ni, Mn L-edge, and O K-edge for NCM811 cathode after cycle with Poly(PEGDA)/LiTFSI electrolyte

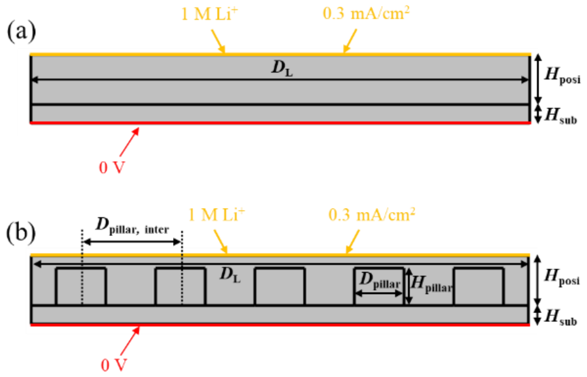

Fig. S38 The geometrical structures and boundary conditions for **a** f-SE electrolyte; **b** p-3DSE electrolyte. Yellow lines indicate the boundary conditions of initial Li^+ concentration and inflow electric current density. Red lines indicate a boundary electric potential of 0 V

Table S2 The values of Rs, and Rct of various Li symmetric cells used different electrolytes cycled after different cycles

Samples	R_s (Ω)	R_{ct} (Ω)
Li/ Poly(PEGDA)-LiTFSI/Li, after 1st cycling	1336.0	1762.0
Li/ Poly(PEGDA)-LiTFSI/Li, after 5th cycling	1092.0	1613.0
Li/ Poly(PEGDA)-LiTFSI/Li, after 20th cycling	288.5	668.1
Li/ Poly(PEGDA)-LiTFSI/Li, after 50th cycling	3378.0	2160.0
Li/f-SE/Li, after 1st cycling	908.3	1556.7
Li/f-SE/Li, after 5th cycling	974.7	1668.3
Li/f-SE/Li, after 20th cycling	888.4	1972.6
Li/f-SE/Li, after 50th cycling	977.7	2111.3
Li/s-3DSE/Li, after 1st cycling	926.7	1538.3
Li/s-3DSE/Li, after 5th cycling	390.8	1178.2
Li/s-3DSE/Li, after 20th cycling	203.9	243.4
Li/s-3DSE/Li, after 50th cycling	280.7	434.2
Li/p-3DSE/Li, after 1st cycling	287.8	435.3
Li/p-3DSE/Li, after 5th cycling	290.5	316.6
Li/p-3DSE/Li, after 20th cycling	199.2	45.7
Li/p-3DSE/Li, after 50th cycling	90.97	26.53

Table S3 The values of Rs, and Rct of various Li/p-3DSE/Li symmetric cells at different temperature

Temperature (℃)	Rs (Ω)	Rct (Ω)
40	114.4	43.2
60	90.97	24.63
80	36.9	24
100	21.2	12.6

Table S4 Comparison of the cycling current density and lifespan of Li symmetric cells with previously reported 3D electrolytes

Samples	Current density (mA cm^{-2})	Overpotential (mV)	Temperature (℃)	Cycle (h)	Refs.
3D LLZOF	0.1	80	50	1000	S1
FPG	0.15	100	60	1400	S2
VPI-ZnO	0.1	40	60	450	S3
Ca-CeO_2	0.1	100	60	1000	S4
Enriched LiFE	0.1	50	50	1600	S5
Aligned PI	0.1	60	60	1000	S6
Al_2O_3-PEO	0.05	170	60	1400	S7
PTF-4EO	0.25	100	30	800	S8
MoO_3-PEO	0.2	121	60	1000	S9

BIT NFs	0.2	75	50	3000	S10
**p-3DSE (This work)**	**0.5**	**46**	**RT**	**2600**	
	**1.0**	**150**	**40**	**1260**	
	**1.0**	**100**	**60**	**1400**	
	**1.0**	**79**	**80**	**1760**	
	**1.0**	**55**	**100**	**2000**	

Table S5 The values of Rs and Rct of Li/p-3DSE/NCM811 full cells with different cycles

cycles (n)	Rs (Ω)	Rct (Ω)
1	3.082	184.958
2	3.074	144.216
5	3.063	134.537
10	3.041	125.889
15	2.950	129.06
20	2.788	125.082
25	2.071	116.949
30	2.056	113.464
35	2.050	108.062
40	2.038	100.63
45	2.030	92.892
50	1.990	85.321

Table S6 Simulation parameters of Li ions diffusion coefficient

Parameters	Value	unit
$D_{\text{f-SE}}$	3.2×10^{-12}	cm^2/s
$D_{\text{s-3DSE}}$	1.6×10^{-11}	cm^2/s
$D_{\text{p-3DSE}}$	6.6×10^{-10}	cm^2/s
D_{cathode}	5.0×10^{-12}	cm^2/s

Supplementary References

[S1] M. Zhang, P. Pan, Z. Cheng, J. Mao, L. Jiang et al., Flexible, mechanically robust, solid-state electrolyte membrane with conducting oxide-enhanced 3D nanofiber networks for lithium batteries. Nano Lett. **21**(16), 7070–7078 (2021). https://doi.org/10.1021/acs.nanolett.1c01704

[S2] Y. Lin, M. Wu, J. Sun, L. Zhang, Q. Jian et al., A high-capacity, long-cycling all-solid-state lithium battery enabled by integrated cathode/ultrathin solid electrolyte. Adv. Energy Mater. **11**(35), 2101612 (2021). https://doi.org/10.1002/aenm.202101612

[S3] A. Tiwari, S. Valyukh, *Advanced Energy Materials* (John Wiley & Sons, 2014).

[S4] W. Bao, L. Zhao, H. Zhao, L. Su, X. Cai et al., Vapor phase infiltration of ZnO quantum dots for all-solid-state PEO-based lithium batteries. Energy Storage Mater. **43**, 258–265 (2021). https://doi.org/10.1016/j.ensm.2021.09.010

[S5] H. Chen, D. Adekoya, L. Hencz, J. Ma, S. Chen et al., Stable seamless interfaces and rapid ionic conductivity of Ca-CeO_2/LiTFSI/PEO composite electrolyte for high-rate and high-voltage all-solid-state battery. Adv. Energy Mater. 2020, **10**(21), 2000049. https://doi.org/10.1002/aenm.202000049

[S6] O. Sheng, J. Zheng, Z. Ju, C. Jin, Y. Wang et al., In situ construction of a LiF-enriched interface for stable all-solid-state batteries and its origin revealed by Cryo-TEM. Adv. Mater. **32**(34), e2000223 (2020). https://doi.org/10.1002/adma.202000223

[S7] J. Wan, J. Xie, X. Kong, Z. Liu, K. Liu et al., Ultrathin, flexible, solid polymer composite electrolyte enabled with aligned nanoporous host for lithium batteries. Nat. Nanotechnol. **14**, 705–711 (2019). https://doi.org/10.1038/s41565-019-0465-3

[S8] F. Wu, Z. Wen, Z. Zhao, J. Bi, Y. Shang et al., Double-network composite solid electrolyte with stable interface for dendrite-free Li metal anode. Energy Storage Mater. **38**, 447–453 (2021). https://doi.org/10.1016/j.ensm.2021.03.020

[S9] H. Li, Y. Du, Q. Zhang, Y. Zhao, F. Lian, A single-ion conducting network as rationally coordinating polymer electrolyte for solid-state Li metal batteries. Adv. Energy Mater. **12**(13), 2103530 (2022). https://doi.org/10.1002/aenm.202103530

[S10] A. Tiwari, S. Valyukh, *Advanced Energy Materials* (2014).

[S11] X. Wang, S. Huang, K. Guo, Y. Min, Q. Xu, Directed and Continuous interfacial channels for optimized ion transport in solid-state electrolytes. Adv. Funct. Mater. **32**(49), 2206976 (2022). https://doi.org/10.1002/adfm.202206976

[S12] Y. Han, Z.W. Gu, Q. Fu, *Advanced Functional Materials* (2015).

[S13] J. Kang, Z. Yan, L. Gao, Y. Zhang, W. Liu et al., Improved ionic conductivity and enhancedinterfacial stability of solid polymer electrolytes with porous ferroelectric ceramic nanofibers. Energy Storage Mater. **53**, 192–203 (2022). https://doi.org/10.1016/j.ensm.2022.09.005
